# Supplementary material for: Compressive stress gradients direct mechanoregulation of anisotropic growth in the zebrafish jaw joint
Source: PLoS Comput Biol. 2024 Feb 8;20(2):e1010940. doi: 10.1371/journal.pcbi.1010940 (PMC10880962; doi:10.1371/journal.pcbi.1010940)
Supplement: S1 Fig — (DOCX) [file pcbi.1010940.s001.docx]

**S1_Fig: Growth simulations from cell-level data**

**
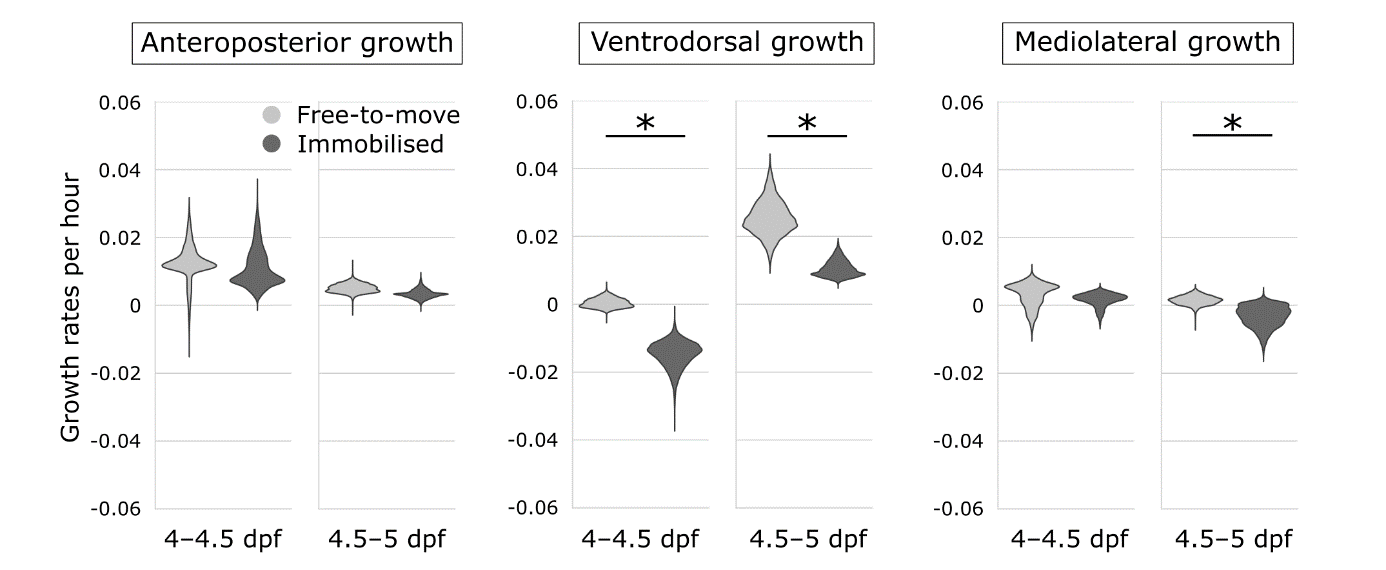
**

**Fig A. Quantitative comparison of ventrodorsal, anteroposterior and mediolateral growth rates between free-to-move and immobilised groups.** These show the growth rates across the ROIs (spatial distribution) and is a quantitative representation of the growth maps which was used to perform statistics. As a reminder, a unique growth map is computed from multiple larvae cell data for each time-window. * indicates significant difference between free-to-move and immobilised means (p<0.05).


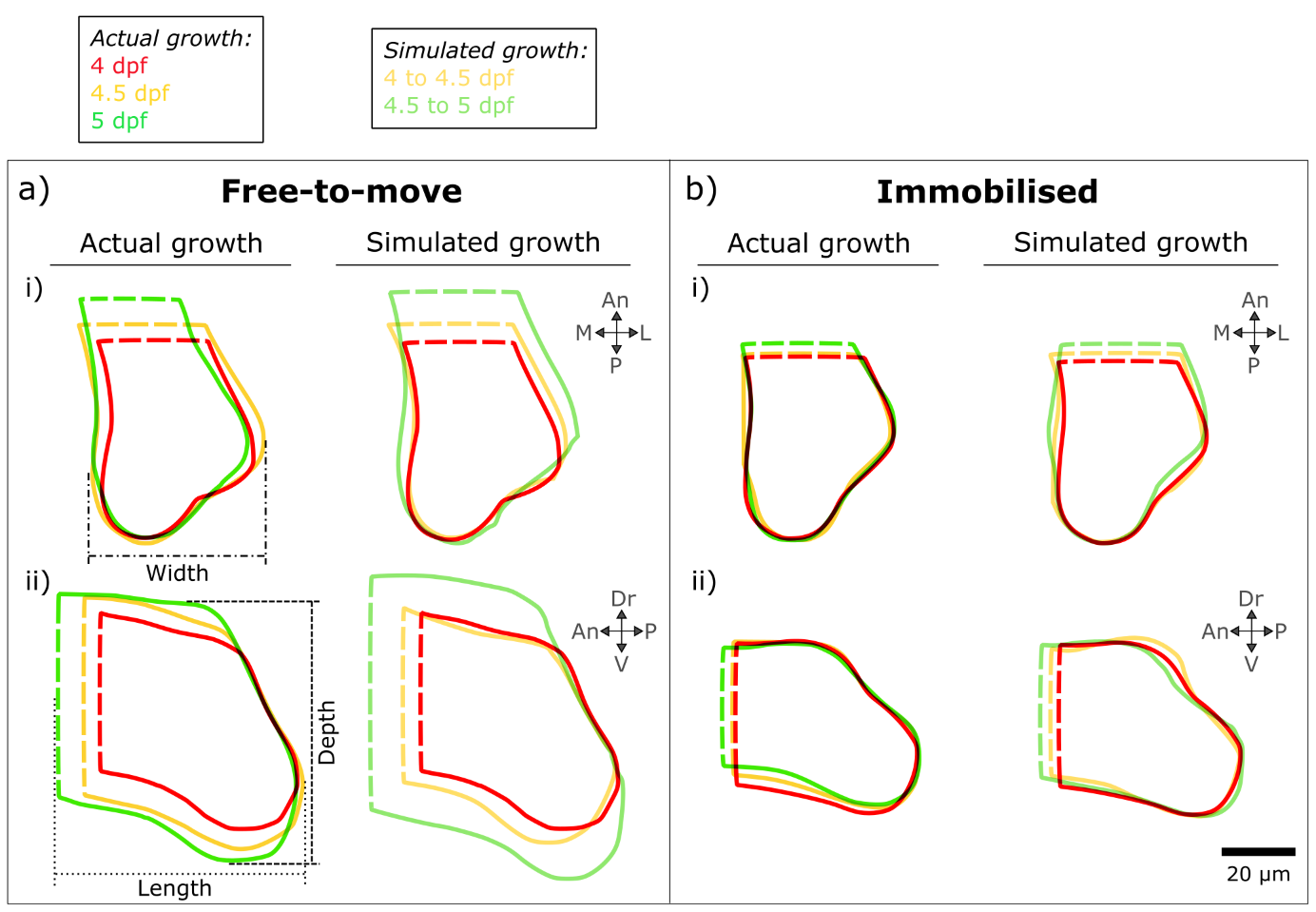


**Fig B.** **Growth simulations correctly predict shape changes in free-to-move and immobilised MC joint elements.** (a, b) Average shape outlines at 4, 4.5 and 5 dpf (left: actual growth) along with simulated growth outlines (right: simulated growth) for each twelve-hour time window (4–4.5 and 4.5–5 dpf) for (a) free-to-move and (b) immobilised larvae in the (i) ventral and (ii) lateral planes. In each time window, the average shape at the starting point was used as the initial shape when simulating growth (e.g. 4 dpf average shape is the initial shape of 4 to 4.5 dpf growth simulation). An: Anterior, Dr: Dorsal, L: Lateral, M: Medial, P: Posterior, V: Ventral.
