# Supplementary material for: Compressive stress gradients direct mechanoregulation of anisotropic growth in the zebrafish jaw joint
Source: PLoS Comput Biol. 2024 Feb 8;20(2):e1010940. doi: 10.1371/journal.pcbi.1010940 (PMC10880962; doi:10.1371/journal.pcbi.1010940)
Supplement: S2 Fig — (DOCX) [file pcbi.1010940.s002.docx]

**S2_Fig: Mechanobiological models from 4.5 to 5 dpf**


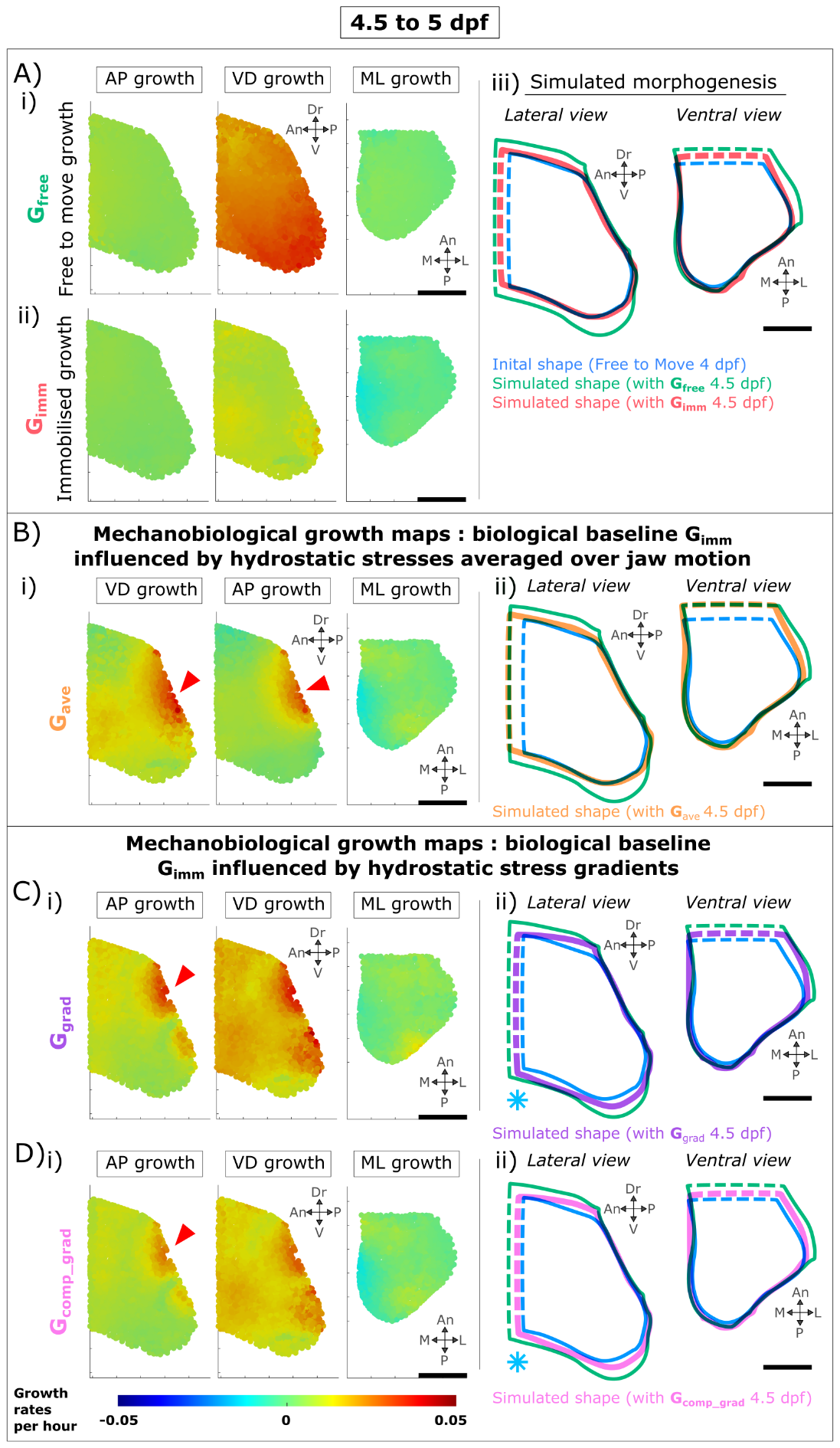


**Mechanobiological simulations of zebrafish larval jaw joint morphogenesis from 4–4.5 dpf incorporating different biological and mechanobiological contributions.** A) Biological contributions to morphogenesis in the absence of movements lead to undergrowth of MC depth and length compared to free-to-move shapes. A-i) Immobilised ventrodorsal (VD), anteroposterior (AP) & mediolateral (ML) growth rates applied to free-to-move 4 dpf shape. A-ii) Free-to-move growth rates. A-iii) Outlines of simulated morphogenesis with immobilised or free-to-move growth rates promoting growth. B) Hydrostatic stresses averaged over jaw motion when used as the mechanoregulatory stimulus fail to simulate physiological jaw joint morphogenesis. B-i) Mechanobiological growth rates with compression promoting growth. Red arrowheads point to local areas of non-physiological elevated growth rates. B-ii) Outlines of simulated morphogenesis. C) Hydrostatic stress gradients as mechanobiological stimulus offer enhanced predictions of jaw joint morphogenesis. C-i) Mechanobiological growth maps in which the biological baseline Gimm is influenced by the hydrostatic stress gradients at peak jaw opening and at peak jaw closure. Red/blue arrowheads point local areas of elevated/reduced growth rates which are not physiological. C-ii) Outlines of simulated morphogenesis. Red arrow shows MC width overgrowth. D) Compressive hydrostatic stress gradients as the stimulus for growth led to the most physiologically correct simulation of jaw joint growth. D-i) Mechanobiological growth maps in which the biological baseline Gimm is influenced by the compressive hydrostatic stress gradients at peak opening and at peak closure. D-ii) Outlines of simulated morphogenesis. Green arrow shows the most physiological MC width as compared to previous simulations. Scale bars are 20 µm. An: Anterior, Dr: Dorsal, L: Lateral, M: Medial, P: Posterior, V: Ventral.
