# Supplementary material for: Compressive stress gradients direct mechanoregulation of anisotropic growth in the zebrafish jaw joint
Source: PLoS Comput Biol. 2024 Feb 8;20(2):e1010940. doi: 10.1371/journal.pcbi.1010940 (PMC10880962; doi:10.1371/journal.pcbi.1010940)
Supplement: S3 Fig — (DOCX) [file pcbi.1010940.s003.docx]

**S3_Fig: Hydrostatic stress fields in the ventral plane**


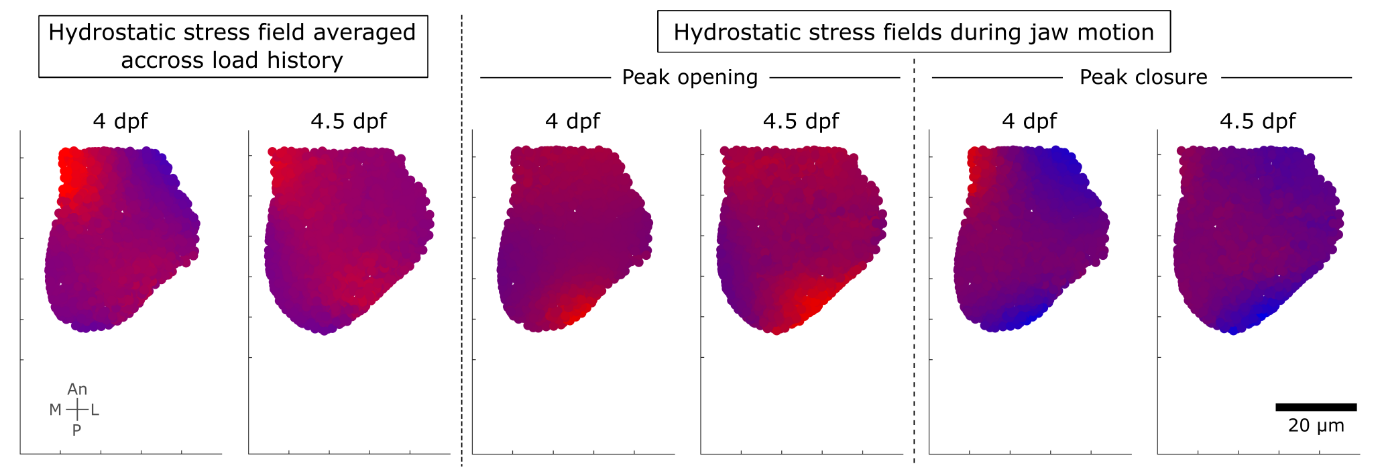


**Ventral views of the hydrostatic stress fields averaged across load history and at peak opening and peak closure.** An: Anterior, M: Medial, L: Lateral, P: Posterior.
