# Supplementary material for: Compressive stress gradients direct mechanoregulation of anisotropic growth in the zebrafish jaw joint
Source: PLoS Comput Biol. 2024 Feb 8;20(2):e1010940. doi: 10.1371/journal.pcbi.1010940 (PMC10880962; doi:10.1371/journal.pcbi.1010940)
Supplement: S4 Fig — (DOCX) [file pcbi.1010940.s004.docx]

**S4_Fig: Jaw joint growth orientations**


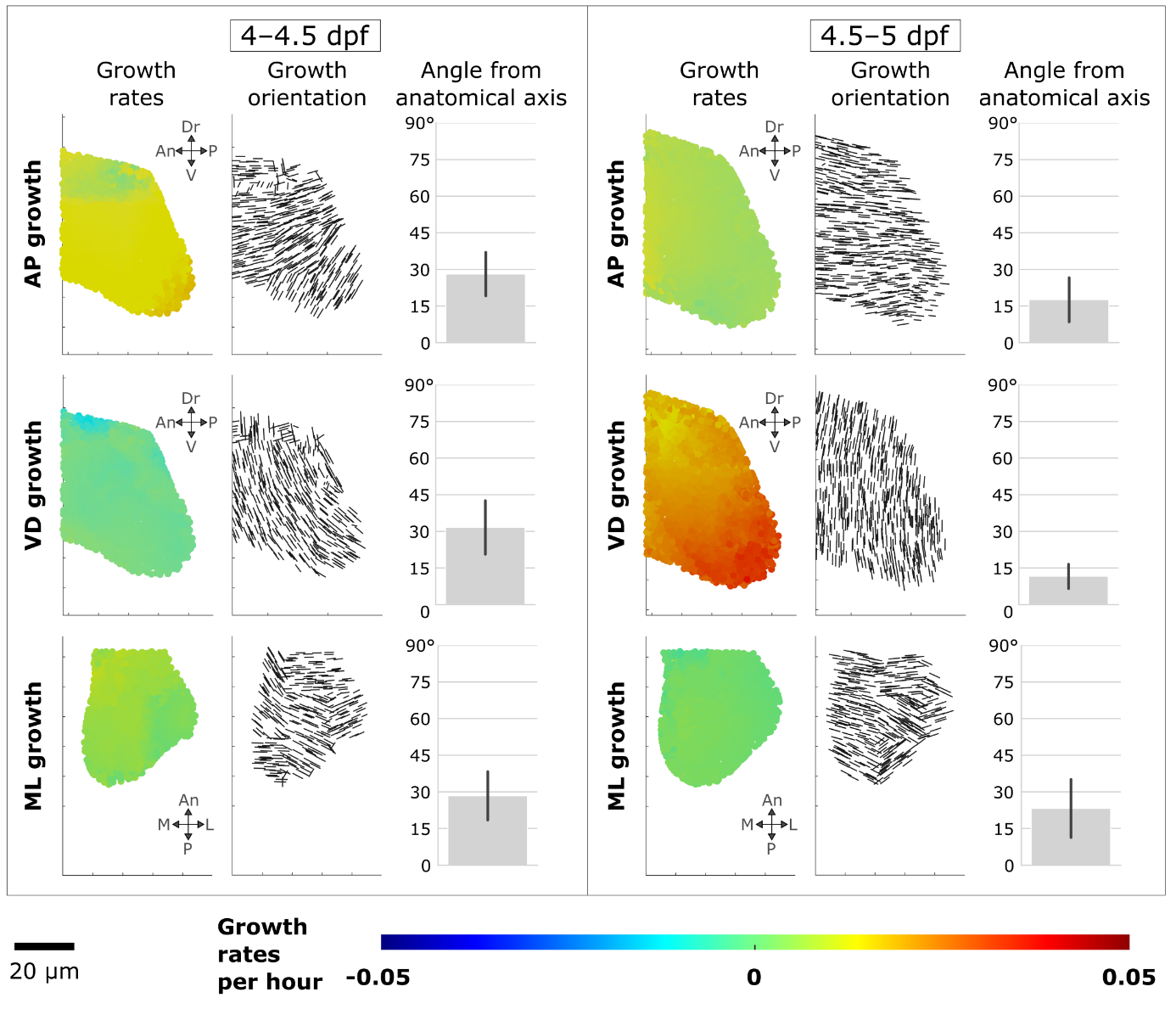


**Fig A. Growth orientations in free-to-move larvae.** Maps showing ventrodorsal (VD)/anteroposterior (AP)/mediolateral (ML) growth rates (colours) and orientations (solid lines) in the MC joint element. Growth orientations correspond to the axis of the local growth ellipsoid (major, median or minor axis). Results are displayed in one section in the mid lateral plane for AP and VD growth and in ne section in the mid ventral plane for ML growth. The angles between the growth orientations and the associated anatomical axis (e.g between VD growth orientations and VD anatomical axis) are shown in bars. An: Anterior, M: Medial, Dr: Dorsal, L: Lateral, P: Posterior, V: Ventral.


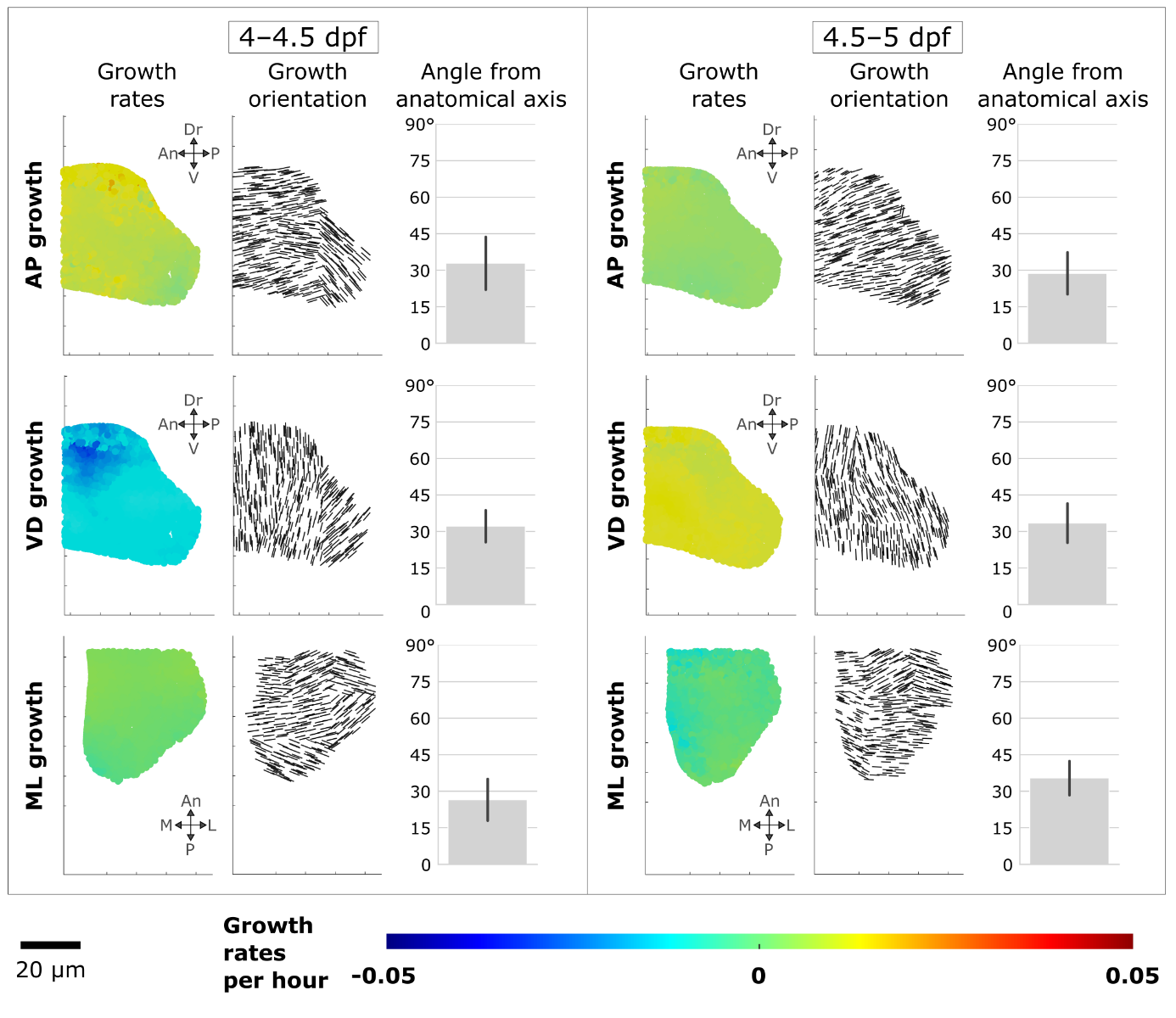


**Fig B. Growth orientations in immobilised larvae.** Maps showing ventrodorsal (VD)/anteroposterior (AP)/mediolateral (ML) growth rates (colours) and orientations (solid lines) in the MC joint element. Growth orientations correspond to the axis of the local growth ellipsoid (major, median or minor axis). Results are displayed in one section in the mid lateral plane for AP and VD growth and in ne section in the mid ventral plane for ML growth. The angles between the growth orientations and the associated anatomical axis (e.g between VD growth orientations and VD anatomical axis) are shown in bars. An: Anterior, M: Medial, Dr: Dorsal, L: Lateral, P: Posterior, V: Ventral.


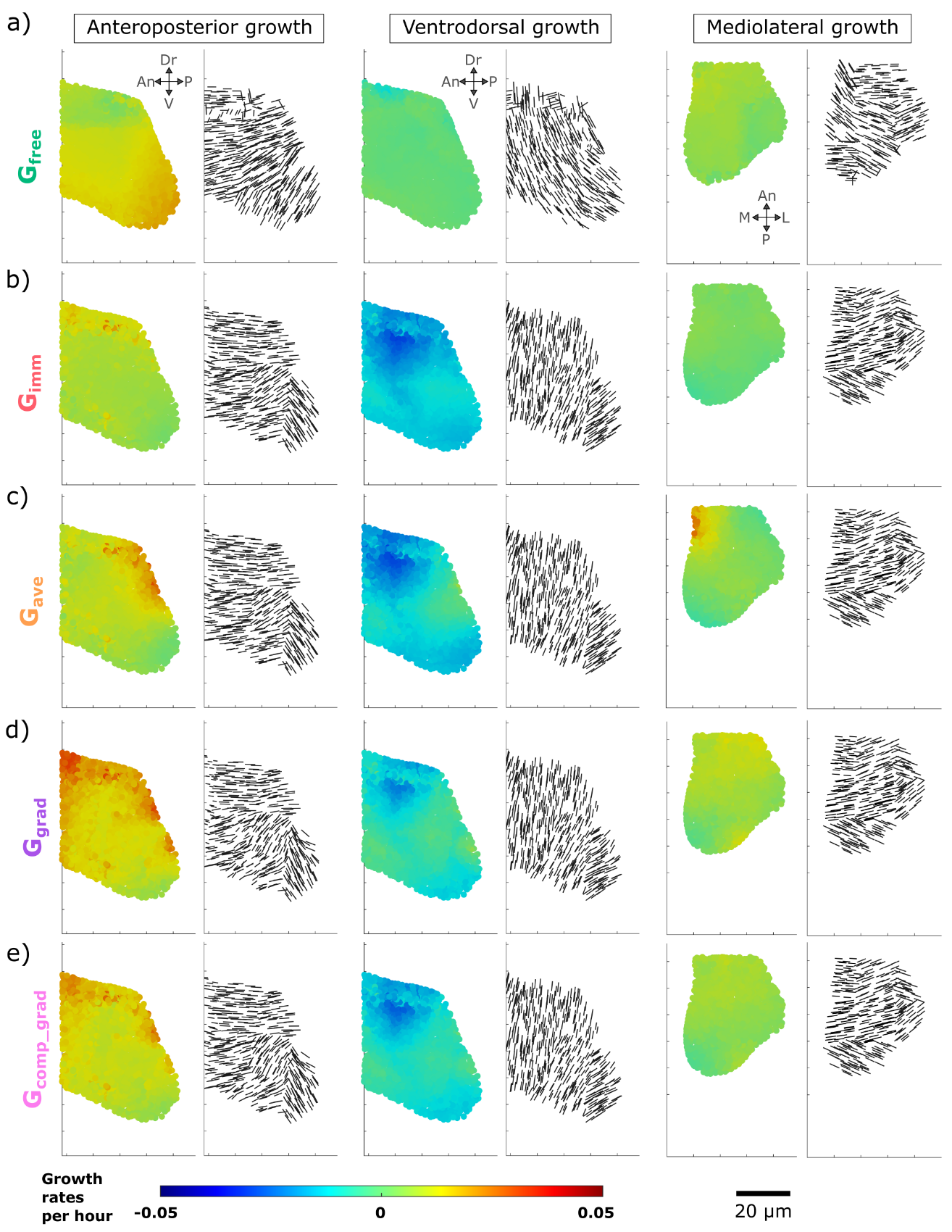


**Fig C. 4 to 4.5 dpf mechanobiological growth maps orientations.** (a) **G_imm_**, (b) **G_free_**, (c) **G_compression_**, (d) **G_dyn_** and (e) **G_dyn_compression_** growth maps from 4 to 4.5dpf in the free-to-move MC joint element. Maps showing ventrodorsal/anteroposterior/mediolateral growth rates (colours) and growth orientations (solid lines). Growth orientations correspond to the axis of the local growth ellipsoid (major, median and minor axes). Results are displayed in one section in the mid lateral plane for AP and VD growth and in ne section in the mid ventral plane for ML growth. Mechanoregulatory growth modulating variables a = 3e9 N^-1^s^-1^ and b = 5e10 m.N^-1^. An: Anterior, M: Medial, Dr: Dorsal, L: Lateral, P: Posterior, V: Ventral.


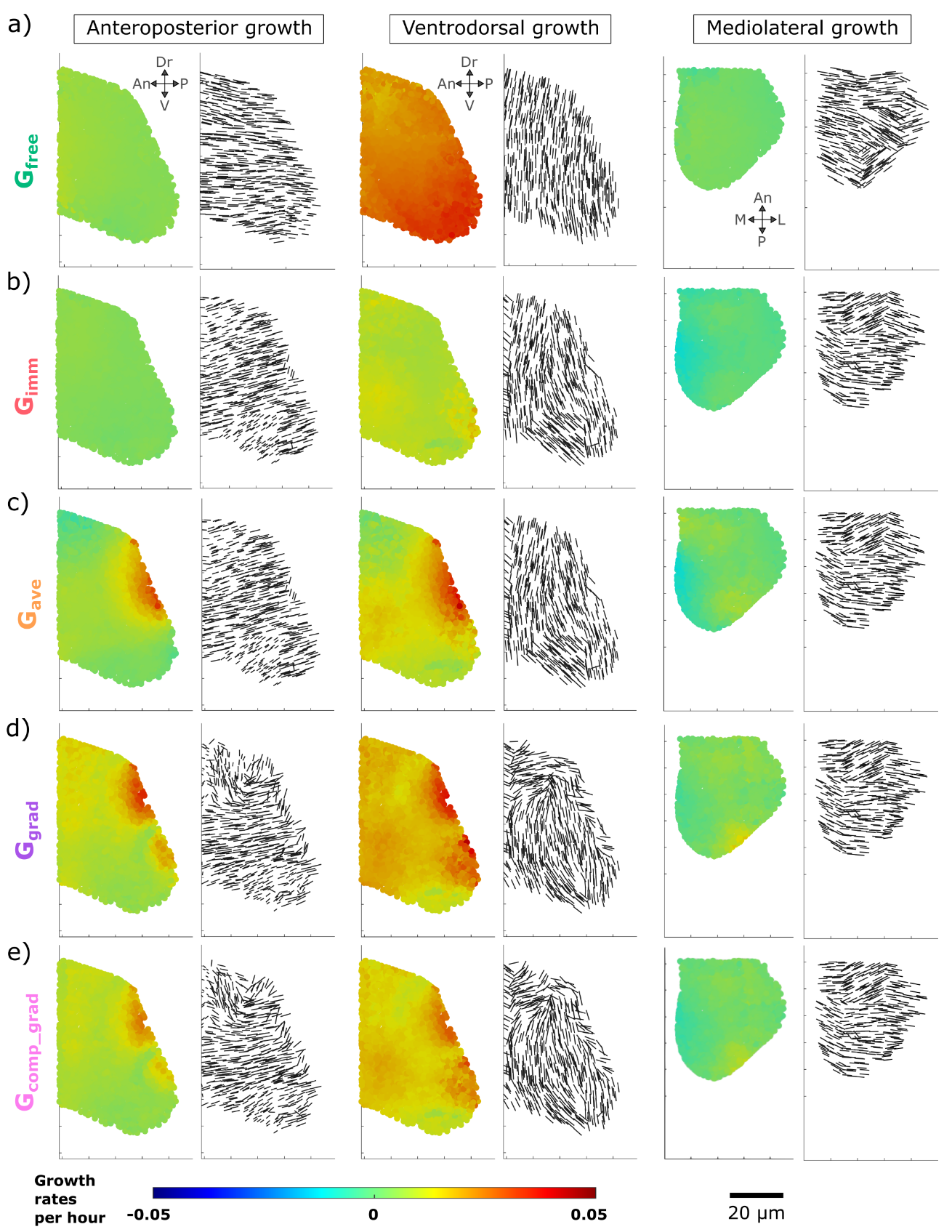


**Fig D. 4.5 to 5 dpf mechanobiological growth maps orientations.** a) **G_imm_**, (b) **G_free_**, (c) **G_compression_**, (d) **G_dyn_** and (e) **G_dyn_compression_** growth maps from 4.5 to 5dpf in the free-to-move MC joint element. Maps showing ventrodorsal/anteroposterior/mediolateral growth rates (colours) and growth orientations (solid lines). Growth orientations correspond to the axis of the local growth ellipsoid (major, median and minor axes). Results are displayed in one section in the mid lateral plane for AP and VD growth and in ne section in the mid ventral plane for ML growth. Mechanoregulatory growth modulating variables a = 3e9 m².N^-1^.s^-1^ and b = 5e10 m^3^.N^-1^.s^-1^. An: Anterior, M: Medial, Dr: Dorsal, L: Lateral, P: Posterior, V: Ventral.
