# Supplementary material for: Compressive stress gradients direct mechanoregulation of anisotropic growth in the zebrafish jaw joint
Source: PLoS Comput Biol. 2024 Feb 8;20(2):e1010940. doi: 10.1371/journal.pcbi.1010940 (PMC10880962; doi:10.1371/journal.pcbi.1010940)
Supplement: S5 Fig — (DOCX) [file pcbi.1010940.s005.docx]

**S5_Fig: Sensitivity analyses of mechanoregulatory growth modulating variables**


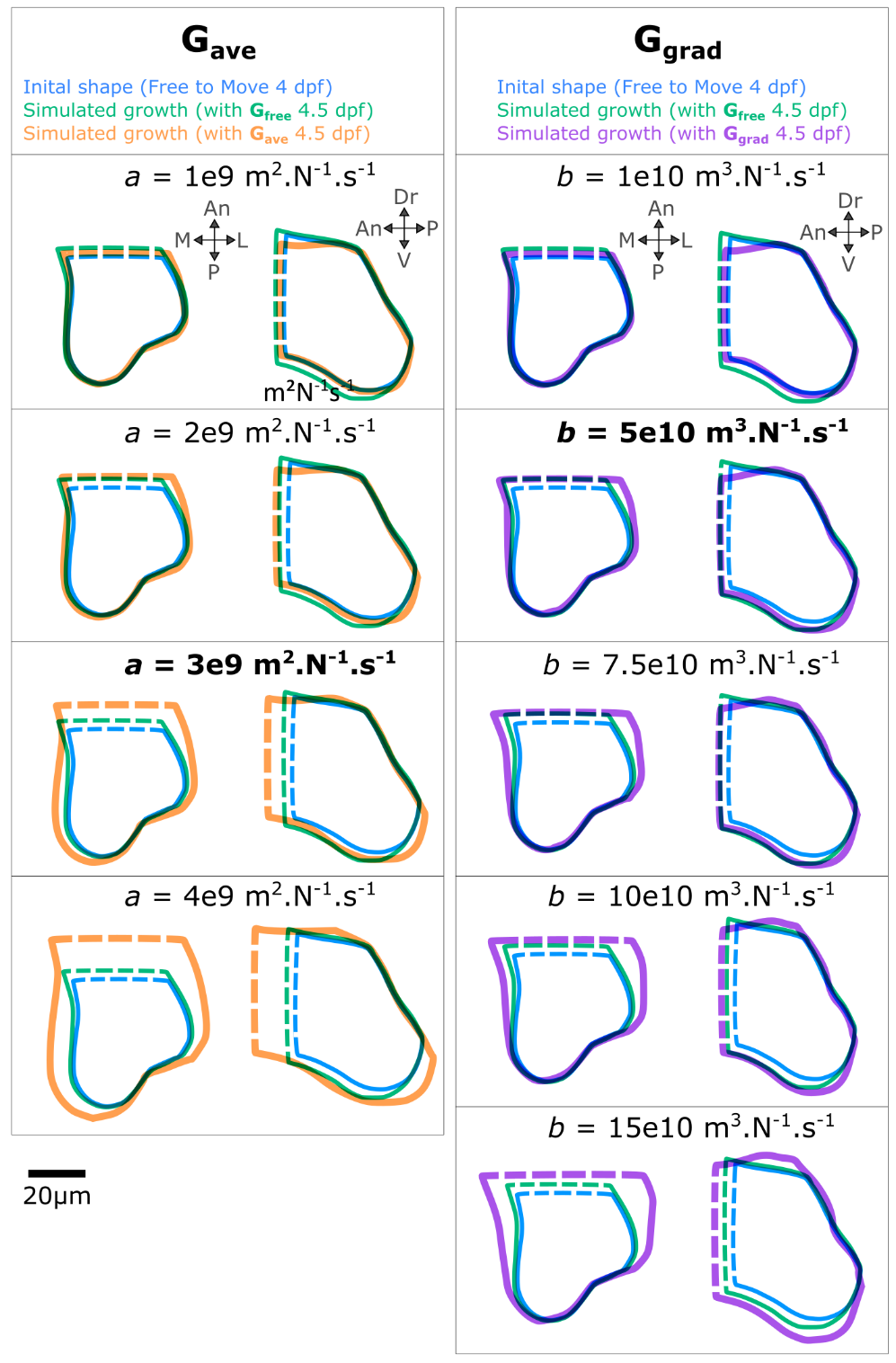


**Sensitivity analyses of** **mechanoregulatory growth modulating variables affecting growth rates from 4 to 4.5 dpf.** Shape outlines of growth simulations using **G_compression_** and **G_dyn_** with the modulating variables *a* and *b* being incrementally increased. The modulating variable enabling physiological growth of the MC depth was chosen and is highlighted in bold. An: Anterior, Dr: Dorsal, L: Lateral, M: Medial, P: Posterior, V: Ventral.
