# Supplementary material for: Compressive stress gradients direct mechanoregulation of anisotropic growth in the zebrafish jaw joint
Source: PLoS Comput Biol. 2024 Feb 8;20(2):e1010940. doi: 10.1371/journal.pcbi.1010940 (PMC10880962; doi:10.1371/journal.pcbi.1010940)
Supplement: S1 Text — (DOCX) [file pcbi.1010940.s006.docx]

**S1_Text: growth rates calculations from cell positional information**

This supplementary data file provides details on how growth rates calculations were performed from cell positional information following the methodology developed by Graner et al. [1].

A cubic grid of side length fifteen microns was superimposed on the aligned joints to divide them into regions of interest (ROIs) as shown in Fig A.a. For each ROI, cells within the ROI’s limits were detected and their adjoining cells were listed. Vectors linking the centroids of adjacent cells were created as shown in Fig A.b. The relative position of cell centroids in an ROI at a timepoint *t* forms a pattern which can be described by a texture matrix *M* calculated from the link vectors $l$ connecting neighbouring cell centroids (equation 1) (Fig A.b) [1].

|  | $M=\left\langle l \bigotimes l \right\rangle$ | ( 1 ) |
| --- | --- | --- |

where $l$ is a link vector connecting two neighbouring cells, $\left\langle\ldots\right\rangle$ denotes averaging taking all links within an ROI into consideration, ⨂ is the outer (or tensor) product, *M* is the texture matrix within an ROI at time point *t*, in a fish. *M* has the dimension of [m²] and, in 3D, is a 3x3 matrix.

Over time the link vectors $l$ may change in length and direction, changing the pattern’s overall geometry (Fig A.b). Graner et al. [1] defined the “statistical symmetrised velocity gradient” (matrix *V* ) as a tool to describe at which rate and in which direction the pattern of the tissue deforms between consecutive timepoints *t* and *t+Δt*. This gradient quantifies local tissue distortions, such that if cells within an ROI grow or intercalate, or if extracellular matrix is built, the distance between cell centroids, and therefore the geometry of the tissue, change (Fig A.b). *V* , set by Graner et al [1], writes as follows (equation 2):


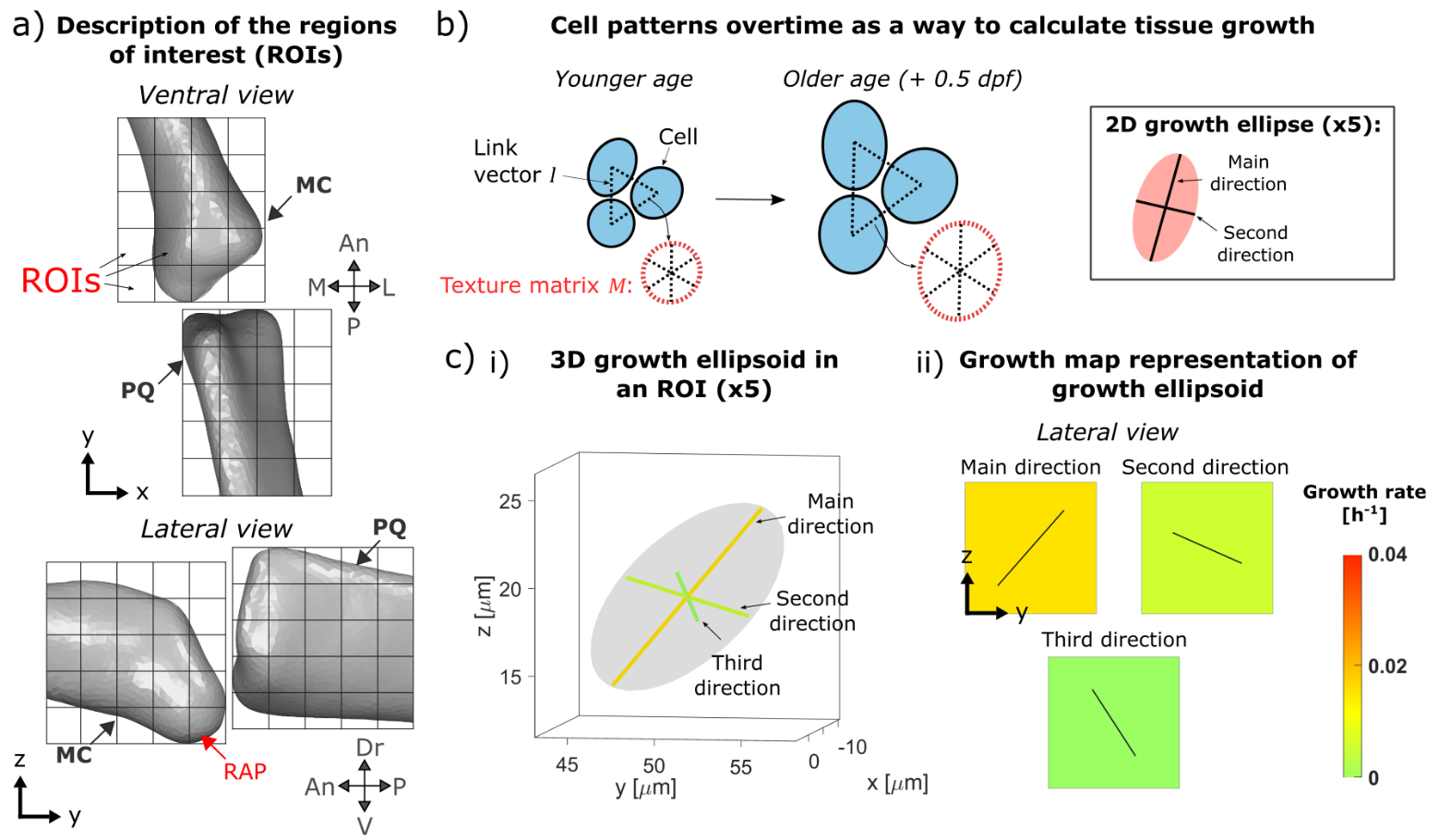


**Fig A.** **Growth rate calculations.** a) A grid marks out the regions (ROIs) of the anterior MC and posterior PQ joint elements in which growth is characterised. Each cube side length is 15µm. b) The position of cells with respect to each other forms a pattern described by a texture matrix M. This pattern evolves overtime which enables local growth characterisation. c) i) The growth rate calculated for each ROI is represented by an ellipsoid with orthogonal axes. ii) The ellipsoid’s radii and the orientation of its axes are used to generate a growth map for each of the ellipsoid’s radii in the lateral plane; growth rate is represented by the square’s colour while the direction of growth is shown by solid black lines in the corresponding square. MC: Meckel’s cartilage, PQ: Palatoquadrate, CH: ceratohyal, RAP: retroarticular process, An: Anterior, P: Posterior, L: Lateral, M: Medial, Dr: Dorsal, V: Ventral.

|  | $V= \frac{W+ W^{T}}{2}= \frac{M^{-1}C+ C^{T}M^{-1}}{2}$ | ( 2 ) |
| --- | --- | --- |

where *T* denotes matrix transposition and intermediate steps *W* and *C* write as:

|  | $C=\left\langle l \bigotimes\frac{dl}{dt} \right\rangle$ | ( 3 ) |
| --- | --- | --- |
|  | $W=M^{-1}C=\left\langle l \bigotimes l \right\rangle^{-1} \left\langle l \bigotimes\frac{dl}{dt} \right\rangle$ | ( 4 ) |

where *M* is the texture matrix within an ROI, $l$ is a link vector connecting two neighbouring cells, $\frac{dl}{dt}$ is the time derivative of a link vector $l$*,* $\left\langle... \right\rangle$ denotes averaging taking all links within an ROI into consideration, ⨂ is the outer (or tensor) product.

$V$ has the dimension of a strain rate [s^-1^] and is a statistical measurement of tissue changes based on discrete objects. In continuum mechanics, $V$ is an equivalent to the velocity gradient’s symmetrical part $\dot{\varepsilon}$, that is, the strain rate [1], as explained below:

|  | $\dot{\varepsilon}= \frac{\nabla v+ \nabla v^{T}}{2}$ | ( 5 ) |
| --- | --- | --- |

where the velocity gradient $\nabla v$ is the spatial derivative of the velocity field, and $\nabla v^{T}$ is its transposed.

Under the affine assumption where the velocity field is considered continuous and linearly varying with position (Fig B), consider a link $l= r_{2}- r_{1}$ ( 6 ) which connects two objects of coordinates $r_{1}$ and $r_{2}$. Its time derivative is:

|  | $\frac{dl}{dt}=v(r_{2})- v(r_{1})$ | ( 7 ) |
| --- | --- | --- |

where $v(\ldots)$ is the velocity of each individual object [1]. Under the affine assumption we have:

|  | $v\left( r_{2} \right) \sim v\left( r_{1} \right)+\nabla v^{T}.(r_{2}- r_{1})$ | ( 8 ) |
| --- | --- | --- |
| that is | $\frac{dl}{dt}\sim\nabla v^{T}.l$ | ( 9 ) |

where $\nabla v^{T}$ is the transposed of the velocity gradient $\nabla v$, $l$ is a link vector connecting two objects of coordinates $r_{1}$ and $r_{2}$, *v(…)* is the velocity of each individual object [1].

From equation 3, the transposed of the matrix *C* is $C^{T}=\left\langle\frac{dl}{dt} \bigotimes l \right\rangle$ ( 10 ) [1]. Therefore, under the affine assumption, using equation 9:

|  | $C^{T}\sim\left\langle(\nabla v^{T}.l) \bigotimes l \right\rangle$ | ( 11 ) |
| --- | --- | --- |
| that is | $C \sim M \nabla v$ | ( 12 ) |

where $\nabla v$ is the velocity gradient, $\nabla v^{T}$ is transposed, *M* is the texture matrix within an ROI, $l$ is a link vector*,* $\left\langle... \right\rangle$ denotes averaging taking all links within an ROI into consideration, ⨂ is the outer (or tensor) product [1].


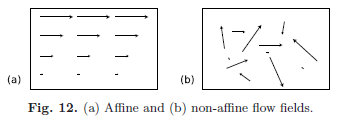


**Fig B.** Affine (a) and non-affine (b) flow fields. From [1]

$W=M^{-1}C$ (equation 4) is then a statistical equivalent of the velocity gradient $\nabla v$ in a discrete description, and *V* (equation 2) is a statistical equivalent to the strain rate (equation 5) [1]. In each ROI, the gradient matrix *V* can be represented by an ellipsoid with orthogonal axes by computing the eigenvalues and eigenvectors of *V*, as illustrated in Fig A.c.i. The orientation of the axes (eigenvectors) and their associated radii (eigenvalues) correspond to the direction and rate of local tissue geometry deformation respectively. Maps of local strain rates (units [s^-1^]) with the associated directions of deformation (main, second and third directions for growth, corresponding to the major, medium and minor axes of the ellipsoid respectively) were generated from each of the three ellipsoid’s axes as shown in Fig A.c.ii. These maps are referred to hereafter as growth maps. Growth maps were calculated for each fish, at each time window. Statistical tests were performed to identify significant differences (p<0.05) between the mean growth rates in each direction for growth in all time windows. Kolmogorov-Smirnov test for normality revealed that groups were not normally distributed (S1 Fig A). Friedman test followed by Wilcoxon signed rank test for paired comparisons with Bonferroni adjustments were performed. For each time point, growth maps were averaged across fish to obtain a unique growth map for each direction for growth. Within each ROI, strain rates that lay outside the interquartile range were removed from the averaging.

1. Graner, F., B. Dollet, C. Raufaste, and P. Marmottant, *Discrete rearranging disordered patterns, part I: robust statistical tools in two or three dimensions.* Eur Phys J E Soft Matter, 2008. **25**(4): p. 349-69.
