# Supplementary material for: Compressive stress gradients direct mechanoregulation of anisotropic growth in the zebrafish jaw joint
Source: PLoS Comput Biol. 2024 Feb 8;20(2):e1010940. doi: 10.1371/journal.pcbi.1010940 (PMC10880962; doi:10.1371/journal.pcbi.1010940)
Supplement: S2 Text — (DOCX) [file pcbi.1010940.s007.docx]

**S2_Text: Material properties characterisation using nano-indentation**

Jaw cartilage material properties in hypertrophic and immature regions were measured in wild type free-to-move and immobilised specimens at 4 and 5 dpf using nano-indentation. The indentation methodology was previously described [1]. Whole larvae were fixed in 4% PFA and stored in 100% MeOH. Prior to nano-indentation, samples were rehydrated to 1 x PBS before being stored in 30% sucrose in PBS. The samples were then submerged in a 1:1 mix of 30% sucrose and optimum cutting temperature (OCT) at room temperature until the samples sunk to the bottom of an Eppendorf tube. Samples were then embedded in fresh 30% sucrose and OCT mix and flash-frozen on dry-ice. Embedded samples were sectioned sagittally at a thickness of 10 µm using an NX70 Cryostat (CryostarTM, ThermoFisher, France). Nano-indentation was performed on sections featuring the Meckel’s cartilage (hypertrophic cartilage), the ceratohyal (hypertrophic cartilage) and/or the jaw joint (immature cartilage) using a Chiaro nanoindenter (Optics11 Life, The Netherlands) as shown in Fig A.a. All measurements were taken in PBS at room temperature. A spherical nano-indentation probe with an 8 µm radius and stiffness of 0.49 N/m was used. Indentation was performed to a depth of 1 µm with velocity of 1 µm/s, and the tip held at a constant depth for 10s (Fig A.b). Nano-indentation was performed across all sections in which the cartilaginous regions were observed, with one measurement collected per region of interest in each section. This was performed for six larvae in each group except in the immobilised 4 dpf group where seven larvae were used. Young’s moduli were estimated using the Hertzian contact model, assuming a Poisson’s ratio of 0.3 (value which was previously used for AFM testing of the larval zebrafish jaw cartilage [1]). The resulting Young’s Moduli were averaged for each fish and for each region across sections. Young’s moduli obtained from measurements taken in the immature regions are shown in Fig A.c.

**
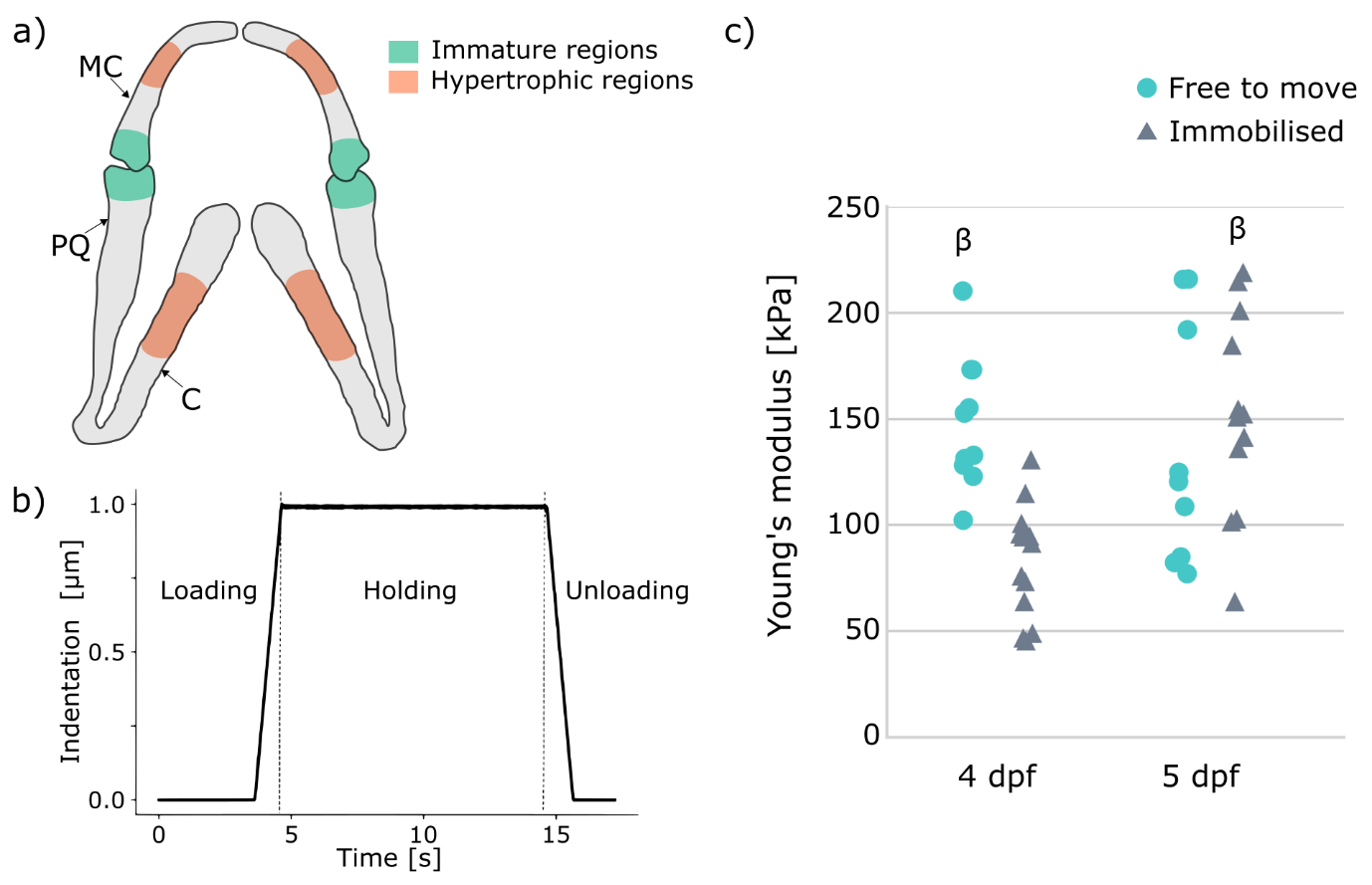
Fig A. Indentation testing.** a) Location of measurements in immature (green) and hypertrophic cartilaginous regions (orange) of larval lower jaw. MC: Meckel’s cartilage, PQ: palatoquadrate, C: ceratohyal. b) Indentation profile consisting of a loading phase during which a depth of 1 µm was reached, a holding phase of 10 s and an unloading phase. c) Young’s moduli of free-to-move and immobilised larvae at 4 and 5 dpf obtained from nano-indentation measurements taken in immature regions. β indicates significant difference (p<0.05) with 4 dpf immobilised group.

Statistical tests were performed in SPSS (IBM Corp. IBM SPSS Statistics for Windows, Version 28.0. Armonk, NY, USA). The Shapiro-Wilks test for normality was performed on each group. To test for significant differences between the hypertrophic and immature regions within each age and larva type group, paired t-tests were performed in groups which were normally distributed, and Wilcoxon signed-rank tests were used in groups which were not normally distributed. No difference was observed between hypertrophic and immature cartilage material properties as shown in Fig B.


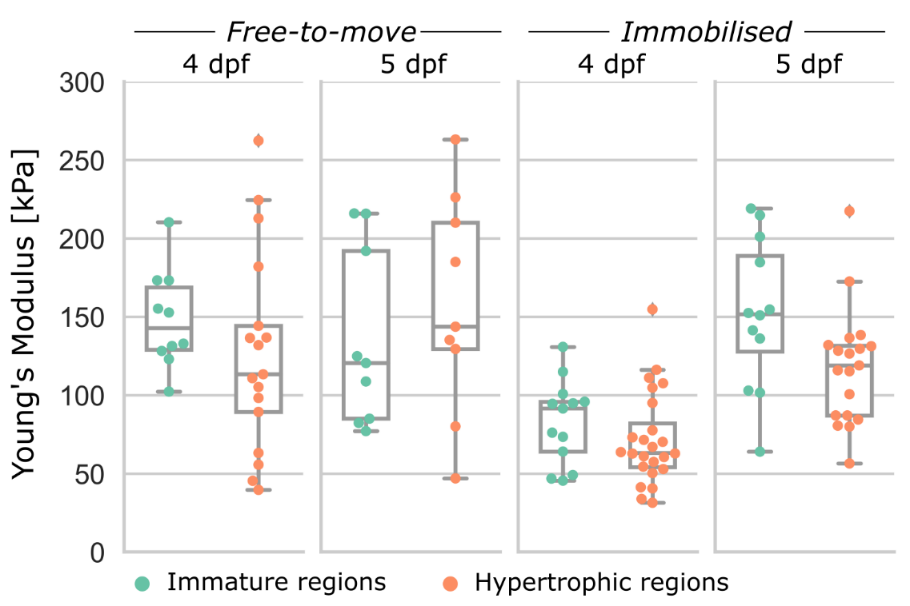


**Fig B. Young’s Moduli calculated from nanoindentation measurements taken in immature and hypertrophic regions in free-to-move and immobilised 4 and 5 dpf larvae.** No significant difference was detected between immature and hypertrophic regions.

Since no significant difference was observed between the hypertrophic and immature regions at any age, tests for significant differences between 4 and 5 dpf free-to-move and immobilised groups were only performed on measurements taken from immature regions. In groups which were normally distributed, One-way Analysis of Variance (ANOVA) with Levene’s test for homogeneity of variance was used. Because the data did not meet the assumption of homogeneity of variance, Games Howell post hoc test was performed to identify groups which differed significantly (significance level 5%). In groups which were not normally distributed, the non-parametric Kruskal-Wallis test was used with Dunn’s post-hoc test and Bonferroni adjustment for multiple comparisons.

No significant difference was observed between 4 dpf and 5 dpf measurements in free-to-move larvae. The average Young’s Modulus when combining data from 4 and 5 dpf in free-to-move larvae was 142.01 kPa. In immobilised larvae, the Young’s Modulus at 4 dpf was found to be lower than for free-to-move specimens with a mean of 82.9 ± 26.6 kPa (Fig A.c). By 5 dpf no difference was observed between immobilised and free-to-move larvae. Results are shown in Table A.

**Table A. Young’s moduli in the immature and hypertrophic regions for free-to-move and immobilised larvae at 4 and 5 dpf.**

|  | **Free-to-move** | | **Immobilised** | |
| --- | --- | --- | --- | --- |
|  | *4 dpf* | *5 dpf* | *4 dpf* | *5dpf* |
| **Immature regions**  **Mean ± SD (kPa)** | 148.23 ± 31.33 | 135.79 ± 56.95 | 82.91 ± 26.61 | 151.96 ± 47.67 |
| **Hypertrophic regions**  **Mean ± SD (kPa)** | 126.55 ± 63.90 | 157.74 ± 69.90 | 71.72 ± 29.60 | 117.82 ± 36.74 |

1. Lawrence, E.A., J. Aggleton, J. van Loon, J. Godivier, R. Harniman, J. Pei, et al., *Exposure to hypergravity during zebrafish development alters cartilage material properties and strain distribution.* Bone Joint Res, 2021. **10**(2): p. 137-148.
