# Supplementary material for: Compressive stress gradients direct mechanoregulation of anisotropic growth in the zebrafish jaw joint
Source: PLoS Comput Biol. 2024 Feb 8;20(2):e1010940. doi: 10.1371/journal.pcbi.1010940 (PMC10880962; doi:10.1371/journal.pcbi.1010940)
Supplement: S3 Text — (DOCX) [file pcbi.1010940.s008.docx]

**S3_Text: Comparison between linear elastic and viscoelastic material properties in jaw movement simulations**

Jaw movements were simulated at 4 dpf using successively linear elastic or viscoelastic material properties. In this appendix, details on how the viscoelastic parameters were determined from nano-indentation measurements are provided. A typical force-indentation curve obtained from nano-indentation measurements is shown in Fig A.a. The material was represented as a spring (elastic modulus E_0_) in parallel to a Maxwell solid (elastic modulus E_1_ and time constant τ_1_) to allow tissue relaxation (Fig A.b). The relaxation function is:

E(t) = E_0_+E_1_e^t/τ1^

E_0_, E_1_ and τ_1_ were obtained from fitting the relaxation function to nano-indentation measurements in Python [1] as shown in Fig A.c, and the average across sample was used. A Poisson ratio ν = 0.3 was assumed (value which was previously used for AFM testing of the larval zebrafish jaw cartilage [2]). In Abaqus CAE, Prony series parameters in the time domain were used:

g_1_ = 1-E_0_/(E_0_+E_1_)

k_1_ = g_1_

Resulting parameters are shown in Table A. Jaw opening and closure were simulated with each step sets to 1s with time increments of 0.1s.


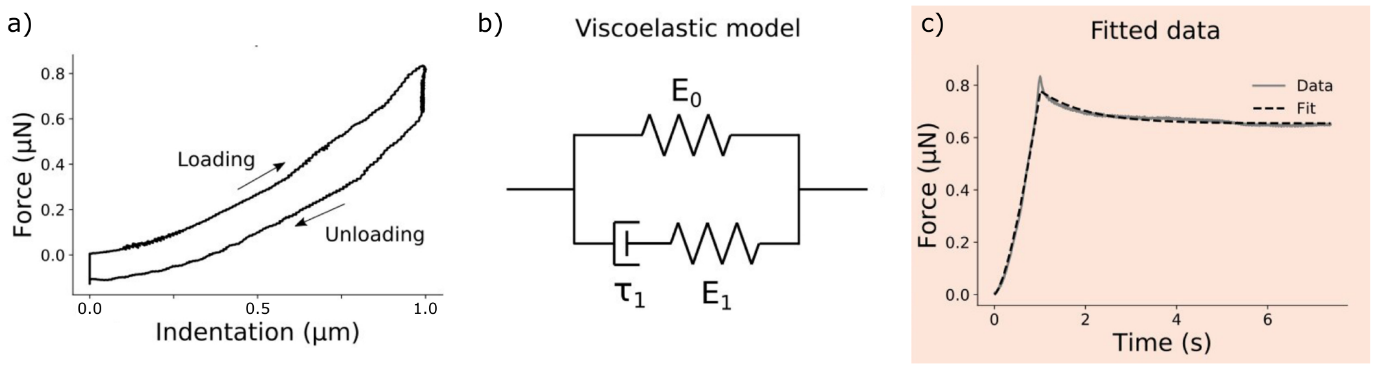


**Fig A: Analyses of nano-indentation measurements.** a) Output force-indentation curve displaying hysteresis. b) The viscoelastic model consists of a spring (elastic modulus E0) in parallel to a spring (elastic modulus E1) and a dashpot (time constant τ1) in series. c) The relaxation function associated with the viscoelastic model is fitted to experimental data to characterise the material’s behaviour during load- and hold-phases. Adapted from [3].

**Table A: Linear elastic and visco elastic parameters used in Abaqus simulations of jaw movement at 4 dpf.**

| **Linear elastic** | **E_Hertz_ [kPa)** | 142.0 |
| --- | --- | --- |
|  | **ν** | 0.3 |
| **Visco elastic** | **E_ins_ [kPa]** | 148.9 |
|  | **ν** | 0.3 |
|  | **E_0_ [kPa]** | 62.7 |
|  | **E_1_ [kPa]** | 51.9 |
|  | **τ_1_ [s^-1^]** | 1.87 |
|  | **g_1_** | 0.45 |

When simulating jaw movement using viscoelastic rather than linear elastic material properties the jaw displacement from peak opening to peak closure increase of 1.3 %. Pressure fields at the level of the jaw however remained similar with subtle changes of the order of magnitude of 10^-14^ Pa. Pressure fields at peak opening and peak closure with both material properties are shown in Fig B.


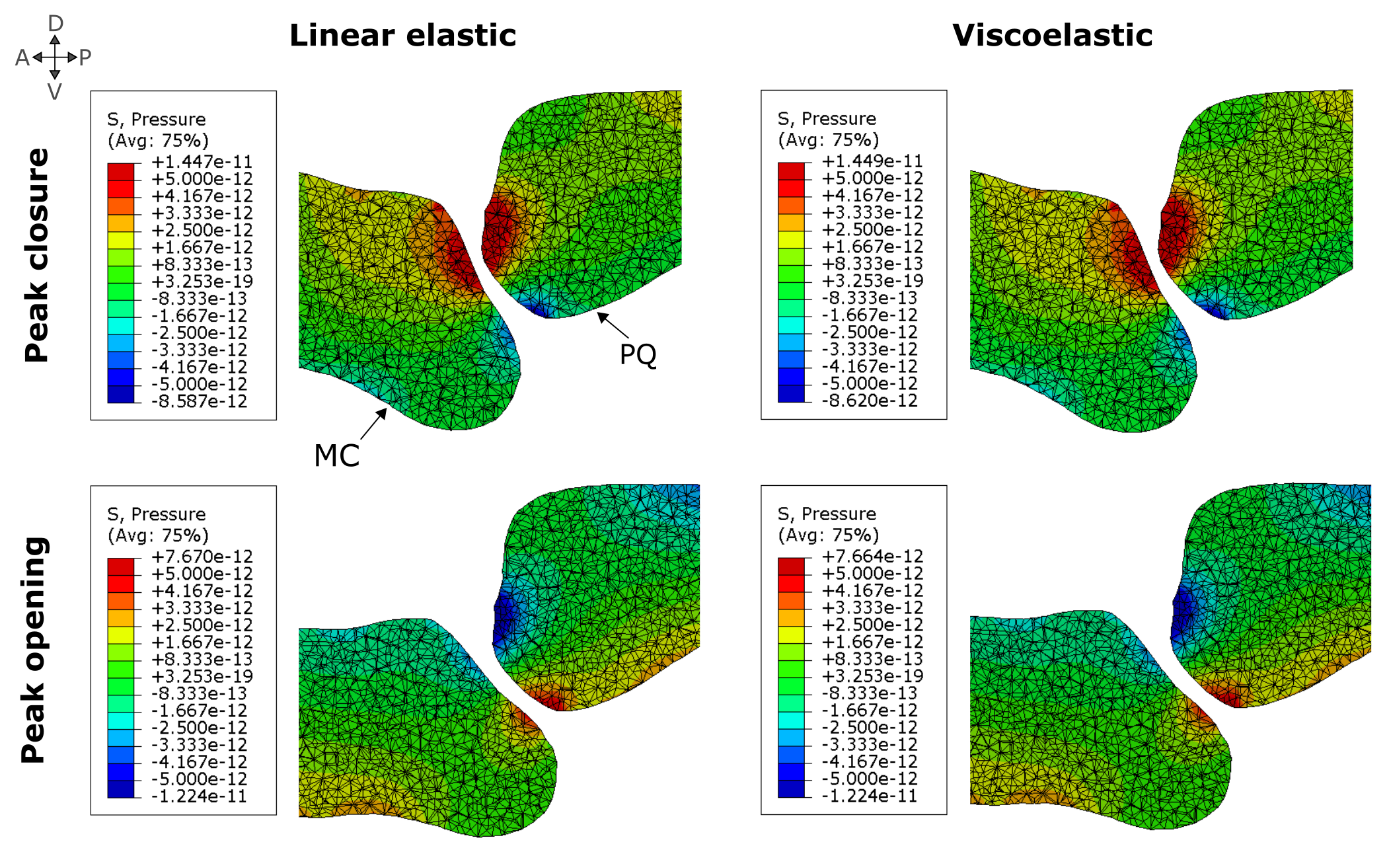


**Fig B: Pressure fields at the level of the joint at peak closure and peak opening when linear elastic or viscoelastic material properties are used.** One section in the lateral plane is shown. A: anterior, D: dorsal, MC: Meckel’s cartilage, P: posterior, PQ: palatoquadrate, V: ventral.

1. Van Rossum, G. and F.L. Drake Jr, *Python reference manual. Centrum voor Wiskunde en Informatica Amsterdam*. 1995.

2. Lawrence, E.A., J. Aggleton, J. van Loon, J. Godivier, R. Harniman, J. Pei, et al., *Exposure to hypergravity during zebrafish development alters cartilage material properties and strain distribution.* Bone Joint Res, 2021. **10**(2): p. 137-148.

3. Levillain, A., S. Ahmed, D.M. Kaimaki, S. Schuler, S. Barros, D. Labonte, et al., *Prenatal muscle forces are necessary for vertebral segmentation and disc structure, but not for notochord involution in mice.* Eur Cell Mater, 2021. **41**: p. 558-575.
