# Supplementary material for: Compressive stress gradients direct mechanoregulation of anisotropic growth in the zebrafish jaw joint
Source: PLoS Comput Biol. 2024 Feb 8;20(2):e1010940. doi: 10.1371/journal.pcbi.1010940 (PMC10880962; doi:10.1371/journal.pcbi.1010940)
Supplement: Attachment — Submitted filename: Answers to reviewers 081223.pdf [file pcbi.1010940.s009.pdf]

The authors would like to express their gratitude to the editor and referees for the constructive comments that have helped improve the quality of the manuscript.
To address the point on data availability, all data underlying the article (confocal images, MATLAB scripts, Abaqus CAE models and simulated shapes) can be accessed on Zenodo at https://doi.org/10.5281/zenodo.7586155.
Below we address the individual points raised by the referees. We have reported each referee's comments (in **bold**) with our responses below and (in *italics*) changes to the text.

Reviewer 1:
This paper examines the role of mechanics in early zebrafish jaw joint growth, combining experiments with computational modeling.

General:
It seems that the basic question is: can you predict dorsal-ventral growth anisotropy with mechanical stimulus? You found that dorsal-ventral growth was greater than other directions and then searched for a mechanical stimulus that would give you this. Is this correct? If so, the paper can be made much more clear throughout.

We initially hypothesised that mechanical stimulus would predict overall growth, but as it turns out, there was minimal growth in two of the three anatomical directions and so our results focus mainly on the mechanoregulation of dorsal-ventral growth. Subsequently, we tested hypotheses on how mechanical stimuli regulate dorsal-ventral growth. We appreciate the succinct summary of the research question, and have tried to simplify the story in the paper as a whole, as illustrated by the extensive tracked changes and as detailed below in response to the specific points.

Title: The title is too overblown. Morphogenesis (joint shape) was not really investigated in this paper. What about "Role of cyclic compression in anisotropic growth in the larval zebrafish jaw"? This brings it back to what was actually studied.

We believe that growth is a type of morphogenesis, and that our work does assess morphogenesis when growth was not uniform in all directions, leading to a different shape at the end of the period studied compared to the start. However, we concede that the title was overstated and have modified it as below.

Changes to Title:
"Compressive stress gradients direct mechanoregulation of anisotropic growth in the zebrafish jaw joint"

This is a lot of detailed work that went into this paper. While it is not necessary to include all the details, you need to include sufficient that we can understand what is going on. For example, what are the loading conditions on your FEA model? Over-complication seems to be a trait of this paper. Sometimes by not describing well, you overcomplicate things. It is currently a very tough paper to grasp. It take a few thorough reads to even understand what you are doing. Let's try to make it more accessible. De-complication is a theme of many of the edits suggested below. You want the reader to skim it once and say "wow! That is really interesting that they found mechanics may drive anisotropic growth. What a clever way to figure this out." Then they read it again for the details of exactly what you did. Right now one gets stuck in the details, but many of the details are not that relevant and relevant details are left out. Specific comments are below. Try to de-complicate and clarify the writing.

We appreciate the feedback and have tried to simplify while including all essential details, and hope that the revisions outlined below have led to a revised manuscript which reads much more clearly. We really do appreciate the huge efforts you have put into helping us to improve the paper.

Specific:
ABSTRACT

Line 21: Change to read "we integrated cell growth data from experiments into a finite element model of zebrafish jaw growth to identify links between mechanical stimuli and anisotropic growth."
Line 32: You imply that "magnitude of compression" is a statically applied load. It is not, it could be cyclic, but you just are capturing the magnitude.
Line 34 "However, the dynamic changes caused by cyclic compression" ….isn't cyclic compression always applied? It is just what you are choosing to look at. All the loading is dynamic.
Line 35: "sizes and shapes of joints were correctly simulated" Unclear that shape was robustly analyzed and size is fairly arbitrary and can be set by a multiplicative constant. Perhaps just stick with "anisotropic growth of the cartilage was correctly simulated".
Line 36: Change the sentence "We conclude…." To "We conclude that the cyclic motion of the jaw joint contributes to anisotropic growth of the cartilage." (or rudiment if it isn't cartilage yet).
Line 38: It is not a "fundamental advance". Change this sentence to read "This study demonstrates the importance of mechanical stimuli in regulating anisotropic growth in early development."

We have almost completely rewritten the abstract to address all of the points raised above. The abstract now reads as follows:

Mechanical stimuli arising from fetal movements are critical factors underlying joint growth. Abnormal fetal movements negatively affect joint shape features with important implications for joint health, but the mechanisms by which mechanical forces from fetal movements influence joint growth are still unclear. In this research, we quantify zebrafish jaw joint growth in 3D in free-to-move and immobilised fish larvae between four and five days post fertilisation. We found that the main changes in size and shape in normally moving fish were in the ventrodorsal axis, while growth anisotropy was lost in the immobilised larvae. We next sought to determine the cell level activities underlying mechanoregulated growth anisotropy by tracking individual cells in the presence or absence of jaw movements, finding that the most dramatic changes in growth rates due to jaw immobility were in the ventrodorsal axis. Finally, we implemented mechanobiological simulations of joint growth with which we tested hypotheses relating specific mechanical stimuli to mechanoregulated growth anisotropy. Different types of mechanical stimulation were incorporated into the simulation to provide the mechanoregulated component of growth, in addition to the baseline (non-mechanoregulated) growth which occurs in the immobilised animals. We found that when average tissue stress over the opening and closing cycle of the joint was used as the stimulus for mechanoregulated growth, joint morphogenesis was not accurately predicted. Predictions were improved when using the stress gradients along the rudiment axes (i.e., the variation in magnitude of compression to magnitude of tension between local regions). However, the most accurate predictions were obtained when using the compressive stress gradients (i.e., the variation in compressive stress magnitude) along the rudiment axes. We conclude therefore that the dominant biophysical stimulus contributing to growth anisotropy during early joint development is the gradient of compressive stress experienced along the growth axes under cyclical loading.

INTRODUCTION

Line 118: One should be very careful linking the stimuli for growth and ossification with the stimuli for cartilage growth. Make it clear that "octahedral shear stress" is for hypertrophy and ossification of the cartilage. Yes, this is growth, but different than what you are looking at in this paper where you hardly have any matrix at the timepoint of interest…we are far away from hypertrophy and ossification.

Agreed: as the focus of this paragraph was really on simulations of cartilage growth, the mention of octahedral shear stress was removed.

Line 123: It is not super convincing that cell-level dynamics contribute critically to the study. Cell-level dynamics provides the mechanism through with dorsal-ventral anisotropic growth

occurs, but you are not directly modeling cell migration, cell proliferation, etc. Be more precise about what you mean "cell-level dynamics" and how it is "necessary".

We intended to make the point that accurate measurements of the cell and matrix activities underlying quantified growth are needed to corroborate hypotheses relating mechanical stimuli to said growth. We have clarified the introductory text as described below.

Page 6, lines 110 - 113,
The importance of measuring accurate cell-level dynamics to truly understand growth is exemplified by prior studies simulating limb bud growth and morphogenesis, in which wildly different theoretical growth patterns led to the exact same final shape [43].

Line 144: Unclear what "magnitude of compression" is from "cyclic compression". Cyclic compression is always applied, you are just looking for a stimulus that matches anisotropic growth. You imply "magnitude of compression" is static and "cyclic compression" is the answer. This does not match what you did: compression is always cyclic in the model, correct? Don't we already know that cyclic compression is critical for cartilage growth? Clarify what is novel.

Correct- we have clarified our terminology replacing "magnitude of compression" with "average stress", "dynamic loading" with "stress gradient", and "dynamic compression"" with "compressive stress gradient". We have completely revised the end of the Introduction to afford more clarity on the steps taken in this paper and expanding on the mechanobiological stimuli we investigated. The novelty of the work is clarified at the end of the Introduction as detailed below.

Page 6 line 128 – Page 7 line 150:
"In this research, we aimed to identify the causal relationship between specific aspects of the biomechanical stimuli arising from embryonic movements and the patterns of joint growth. We first quantified the effects of immobility on zebrafish larval jaw joint morphogenesis from 4 to 5 days post fertilisation, finding that the normal level of growth anisotropy- in which the joint grows primarily in the ventrodorsal axis- was largely eliminated by larval immobility. We then quantified the dynamics of growth underlying normal or immobilised growth by tracking individual cells over the developmental period studied, and found that growth rates were most diminished by larval immobility along the ventrodorsal axis. Next, we implemented a mechanobiological simulation of jaw joint morphogenesis and validated that simulations using the tracked cell activities for free to move and immobilised larvae as the inputs for growth predicted the observed shapes correctly. Finally, we implemented simulations in which immobilised growth rates were applied to control joint shapes (serving as the baseline biological contribution to growth), supplemented by a mechanobiological component of growth in order to test hypotheses relating different types of mechanical stimuli to joint morphogenesis. Using this methodological approach, we tested which mechanical stimuli arising from jaw movements are most likely to direct mechanoregulated growth anisotropy in the zebrafish jaw joint. Based on the quantified patterns of biophysical stimuli resulting from jaw movements, we selected three distinct mechanobiological stimuli for separate incorporation into the simulation; namely a) average stress, b) stress gradient along the growth axes (defined as how steep the variation in magnitude of compression to magnitude of tension between local regions over the loading cycle) and c) compressive stress gradient along the growth axes (the steepness of the variation in compressive stress magnitude), finding that organ-level gradients of compressive stress are likely to be a major stimulus for mechanoregulated anisotropic growth in the zebrafish jaw joint, while tension is probably not a key contributor."

RESULTS
Line 152: "no significant differences in shape" Are you really studying morphogenesis? Or just anisotropic growth?

As outlined above, we consider that anisotropic growth certainly constitutes a type of morphogenesis, as some underlying mechanism or mechanisms are controlling growth in one direction preferentially over the others. While there were no significant differences found in the

specific shape measurements performed (length, width, depth) trends over growth are clearly visible in the qualitative and quantitative data. The lack of significance could be due to the relatively small sample size, variation between animals, or both of these factors. We have modified the text to highlight the three measurements assessed for statistical significance.

Page 8, lines 155–172
"The shapes of Meckel's cartilage (MC) joint elements (shown in Fig 1A and B) in larvae immobilised from 3 dpf and from free-to-move larvae (controls) were measured at 4, 4.5 and 5 dpf. While there were no significant differences in MC length (anteroposterior growth), depth (ventrodorsal growth) or width (mediolateral growth) between timepoints within the free-to-move and immobilised groups, the free-to-move larvae exhibited higher increases of MC length and depth over the whole timeframe compared to immobilised larvae as seen with average shape outlines in Fig 1Cb, d. MC length increased by approximately 37% from 4 to 5 dpf in the free-to-move larvae compared to an average increase of 15% in the immobilised larvae over the same timeframe, with a significant decrease in MC length in the immobilised larvae at 5dpf (Fig 1D). Immobilised MC depth remained almost constant from 4 to 5 dpf whereas the average free-to-move MC depth increased over the same timeframe (43% increase in free-to-move larvae, compared to 6% decrease in the immobilised larvae) (Fig 1Cb, d and Fig 1D). MC depth was significantly decreased compared to the free-to-move larvae at 5dpf (Fig 1E). The average free-to-move MC width at the level of the joint increased slightly over the investigated timeframe (11% increase) whereas the immobilised MC width remained almost unchanged over time (4% increase; Fig 1Ca, c and Fig 1D). However, there were not significant differences between the free-to-move and immobilised groups for MC width. Therefore, growth of the depth of the MC (i.e., ventrodorsal growth) was most severely affected by the absence of jaw movements, followed by MC length, with MC width least affected."

Results: The authors have beautiful 3D data that they shrink down to simplified 2D line drawings. They are encouraged to think about how they might better represent the 3D growth.

Thank you for the suggestion- we have incorporated 3D views into Figure 2, 4 and 7.

Figure 1D: The results showing length, depth, and width demonstrates the anisotropy. Do you need cell-level dynamics data to determine that from 4-5dpf, it grows a little in length, a lot in depth, and not at all in width. Can't a ruler do this?

As already mentioned above, work by Morishita et al., 2014 demonstrated that it is possible to obtain the same shape with dramatically different patterns of growth, and therefore, without actually quantifying what is giving rise to the growth, we cannot test hypotheses relating local-level stimuli to local-level growth events. We have clarified the need for cell-level analyses in the text as outlined below.

Page 10, lines 187 - 190
Given that different patterns of growth may result in the same shapes, without being representative of the true underlying biological processes [43], growth dynamics were quantified by tracking individual cells from 4–4.5 and from 4.5–5 dpf, following the methods described below and in [47] to characterise and visualise the actual patterns of growth which give rise to the observed organ-level shape changes.

Figure 2:
A) is very helpful for orientation.
B) does not add much to what we already know from Figure 1. The model does what you tell it to. Is this necessary to show? Perhaps move this figure to Supplementary.

Figure 2 represents the growth quantified through cell tracking, completely unrelated, as yet, to the FE model. Therefore, the data in Figure 2 provides insight into the biological activity underlying the morphology results presented in Figure 1. We have modified the Figure contents to make the distinction between the morphological data, and the tracked cell activity data clearer.

Unclear why the outlined shape does not change for mediolateral growth. How can you see it in the dorsal-ventral/anterior-posterior plane? Why not show a plane that actually has the mediolateral direction in it? The shape of your outline will change.

The relevant sections of the Results has been rephrased for clarity, and the Figures have been rearranged and honed to help the reader. Mediolateral growth is now shown in a ventral plane.

E) Unclear what the violin plots are showing. How many data points do you have here? Are these the growth rates of ROIs? Again, doesn't Figure 1 show the same information? Is this an average or a representative sample? It is a shame that the authors have to shrink beautiful 3D data onto a 2D plane. Are there better ways to represent the 3D data?

The violin plots are the growth rates of ROIs, used to show where there are (and aren't) significant change in growth rates between free to move and immobilised. This is an average across multiple fish. The violin plots have been moved to supplementary data with additional explanations in the captions.

*"Fig S1.1. Quantitative comparison of ventrodorsal, anteroposterior and mediolateral growth rates between free-to-move and immobilised groups. These show the growth rates across the regions of interest (spatial distribution) and provide a quantitative representation of the growth maps in Figure 2. A unique growth map is computed from multiple larvae cell data for each time-window. * indicates significant difference between free-to-move and immobilised means (p<0.05)."*

Paragraph starting in 175. This says we measured anisotropic growth and modeled it to ensure it gave us what we wanted (i.e. we could grow a model by the growth rates calculated and obtain the "right result"). This is all fairly arbitrary, isn't it? Because you are not looking for changes in shape, in which patterns of high growth may be regional, but looking for anisotropic growth, why is magnitude so interesting? You can tune it to give you the magnitude you want. There are surely enough constant multipliers in your model to do this. I think the entire paragraph can be deleted with little lost. If it adds something more than Figure 1, make it clear what is new that we don't know from Figure 1.

The paragraph which started in 175 described results from two distinct experiments, the first being the quantification of growth rates measured from the tracked cell activities, and the second being implementation of these growth rates into a finite element simulation to validate
that we could successfully implement a growth simulation in which accurately measured biological activity would translate to changes in size and shape which matched the reality. We have now split up the two sets of results as detailed below.
It is important to note that at the validation stage of the simulation, the data and quantification of magnitude come of the cell-level data directly with no tuning. This has been clarified in the text as detailed below.

Section named "Computational Simulations of Growth" (Page 12) encapsulates the set of results demonstrating the validation of the simulation using the cell-level data directly. The section following that ("Changes in load patterns during jaw motion stimulate jaw joint growth anisotropy" starts Page 14) encapsulates the set of results in which mechanical stimuli were incorporated to our simulations.

Page 12, lines 212 - 214
"To validate the simulation, we first verified that simulations using the tracked cell activities for either free-to-move or immobilised larvae as the inputs for growth would predict the observed shapes correctly."

Line 206: Is Gimm the average of the immobilized growth rates?

G_{imm} is the growth map determined from cell-level tracking of multiple immobilised larvae and applied to the free to move shape, as clarified in the text as detailed below.

Page 14, lines 257–260
*"We implemented simulations in which immobilised growth rates (named **G_{imm}**) were applied to control joint shapes (serving as the baseline biological contribution to growth), supplemented by a mechanobiological component of growth."*

Line 210: Can we rename the stimuli: 1) average hydrostatic stress; 2) peak hydrostatic stress gradient; 3) peak hydrostatic compressive stress gradient? If you don't like these names, determine better, more concise names for the three stimuli. "Dynamic" and "time evolution" don't see appropriate since loading was dynamic for all of them. If anything "average" captures more the dynamic than analyzing just at peak.

Agreed: for brevity we went with "average stress", "stress gradient" and "compressive stress gradient" while being clear in the text that we are always referring to hydrostatic stress, and also that the gradients are always peaks rather than averages.

It is unclear why you would average hydrostatic stress if you know the cartilage is in bending; the average cancels out any dorsal/ventral differences. This stimulus can be removed from the paper. It is not clear why it was chosen. Other papers that have looked at "average stimulus" are not examining bending conditions, rather multiple compressive loads.

The reviewer is correct that averaging cancels out dorsal/ventral differences. However, we feel it is justified to leave this aspect of the work in, firstly because in many FE and mechanobiological stimulations (including from our own group) [33,35,37,38,39], average stress values are computed or used, and therefore it provides a useful insight to be able to demonstrate that the average stress is not a good candidate biophysical stimuli. Secondly, it is valuable to be able to demonstrate that the system we have developed is capable of adequately testing a hypothesis, or in other words, having an incorrect prediction of growth provides more credence to the predictions of work which do actually match the reality.

Figure 2 BCD and Figure 3A present the growth planes in different orders. Unnecessarily confusing. It is also not clear why these are not the same. Clarify.

Growth plane order within the figures has been made consistent.

Figure 1C has free-to-move (control) on the left and immobilized on the right. Figure 2B has free-to-move on the top and immobilized on the bottom. Figure 2A has immobilized on the top and free to move on the bottom. This is all unnecessarily confusing. Be consistent in the layout so we are clear what we are looking at.

The figures have been revised to try to be as consistent as possible.

Shouldn't the outlines in 3Aiii match the outlines in 1c?

The outlines in Figure 3 are all based on simulations using the various growth rates while the outlines in Figure 1 are from average real joints. If our simulations using the measured growth for either free-to-move or immobilised growth rates were 100% accurate then we would see the exact same outlines in Figure 3 and Figure 1. However, they are quite close. We have clarified the correlation between the predicted and real growth in the text provided below.

Page 12: lines 212–231
A computational simulation of MC growth was implemented using finite element (FE) methods. To validate the simulation, we first verified that simulations using the tracked cell activities for either free-to-move or immobilised larvae as the inputs for growth would predict the observed shapes correctly. Growth in free-to-move and immobilised zebrafish jaws was simulated for each 12-hour interval time window (4–4.5 and 4.5–5 dpf) following the methodology previously published [47] and described in the Methods section. The outcomes for each simulation type were consistent between the two time-windows and therefore the results for 4–4.5 dpf are provided in Figure 3, and the results

from 4.5–5 dpf in Supplementary Figure S2. Growth was simulated in both scenarios using the free-to-move shape as a starting point. When growth was simulated using the measured free-to-move growth rates (as shown in Fig 3Ai, Fig S2Ai) applied to the starting shape, there was a noticeable increase in depth of the rudiment as evidenced with the expansion of the green outline as shown in Figs 3Aiii/S2Aiii. However, when the measured immobilised growth rates were applied (as shown in Figs 3Aii/S2Aii), no increases in depth were observed (red line in Figs 3Aiii/S2Aiii). Therefore, our computational simulation of growth was able to replicate the most important feature found in the experimental data, which is the lack of MC depth increase in the immobilised larvae as compared to the free-to-move larvae. Furthermore, the simulation was also able to replicate the greater length increases in the free-to-move larvae compared to the immobilised larvae, with length increases more pronounced when using the free-to-move growth rates ($\boldsymbol{G_{free}}$*, green outlines; Figs 3A-iii and S2A-iii) than when using the immobilised growth rates (*$\boldsymbol{G_{imm}}$*, red outlines; Figs 3A-iii and S2A-iii). An overlay of the simulated shape outlines from 4 dpf to 5 dpf is shown in Fig S1.2.*

As in all figures, you cannot show ML growth in an AP/DV plane. Show an appropriate cross section.

Figures have been updated accordingly.

Rename stimuli in B, C, D.

All stimuli have been renamed.

What you are trying to do is match the orange and the green in 3bii, correct? Are 3C and 3D different? They look very similar. Rename G_dynm as all loads are dynamics (as opposed to static).

They indeed look very similar, one with tension, the other with only compression, showing that including tension or not doesn't really make a difference. We have rewritten the relevant sections of text as described below.

Page 18, lines 321–338
Finally, we implemented simulations which included only the gradient to and from compression without considering the influence of hydrostatic tensile stresses. Spatial compressive hydrostatic stress gradients along the three anatomical axes at peak jaw opening and at peak jaw closure/over the opening and closing cycle were calculated and combined with the biological baseline G_{imm} to generate a new mechanobiological growth map called G_{comp_grad}. The G_{comp_grad} map was more similar to the physiological growth map G_{free} than the G_{grad} map, with the G_{comp_grad} map having less pronounced "hot spots" of growth in the AP axis than the G_{grad} map as shown with arrows in Fig 3Di. The growth simulated using G_{comp_grad} was very similar to physiological growth (G_{free}) and also to growth simulated using G_{grad} where both tension and compression were included, particularly from 4.5–5dpf (Fig S3). However, from 4–4.5 dpf, MC width increases were more physiological when only compressive stresses were considered (G_{comp_grad}) compared to when tensile stresses was included (G_{grad}), (green arrow in Fig 3D-ii). Therefore, while removing the dynamic tensile components of hydrostatic stress from the mechanobiological growth algorithms did not have dramatic effects on the resultant shapes, simulations using the gradient of hydrostatic compressive stress lead to the shapes which most closely resembled the physiological shapes under normal movements. Taken together, these results indicate that the application of cyclic compressive loads is likely to be a major stimulus for mechanoregulated anisotropic growth in the zebrafish jaw joint, while tension is probably not a key contributor.

Line 268 It is unclear why you chose to compute stress gradients rather than directly computing the time difference between stress maps.

We wanted to mathematically describe the fact that one side was in compression and the other one in tension, rather than just the change at the location ignoring the surrounding stresses. We clarified this in the text provided below.

Page 17, lines 303–308

We next investigated whether the gradient between tension and compression in the dorsal and ventral aspects of the rudiment over the opening and closing cycle (as shown in Fig 4B) could promote growth along the rudiment's depth and thus influences growth anisotropy. The spatial hydrostatic stress gradients over jaw opening and closing along the three anatomical axes were calculated at peak jaw opening and closure and incorporated to $\boldsymbol{G_{imm}}$ *to generate a new mechanobiological growth map called* $\boldsymbol{G_{grad}}$ *as shown in Figure 3C-i..*

Page 18, lines 321–325

Finally, we implemented simulations which included only the gradient to and from compression without considering the influence of hydrostatic tensile stresses. Spatial compressive hydrostatic stress gradients along the three anatomical axes at peak jaw opening and at peak jaw closure were calculated and combined with the biological baseline $\boldsymbol{G_{imm}}$ *to generate a new mechanobiological growth map called* $\boldsymbol{G_{comp\_grad}}$*.*

Line 290: Remove language referring to "dynamic shift". This is not good terminology.

Any mention to this terminology was removed.

DISCUSSION

Line 366: perhaps not a fundamental advance. …just an understanding.

This was accordingly updated

Line 312: It is not clear that you have definitively shown cyclic compression rather than cyclic stress.

The whole first paragraph of the Discussion was rephrased as described below.

Page 19, lines 344–363

"Growth dynamics calculated from tracked cells data confirmed that growth rates were most diminished along specific anatomical axes when jaw movements were absent, verifying the anisotropic effects of mechanical loading on jaw joint morphogenesis. We constructed a mechanobiological simulation of jaw joint morphogenesis which was capable of replicating physiological growth patterns for both normal and absent movements based on tracked cell activities. We selected three potential biophysical stimuli as the main drivers for mechanoregulation of growth anisotropy, based on a finite element analysis of the stresses arising from jaw opening and closing. These three stimuli were stress averaged over jaw motion, the gradient from compressive to tensile stresses over the opening and closing cycle, and the gradient to and from compressive stresses over the loading cycle (without the tensile component). Growth rates proportional to each of these stresses were combined with the baseline "biological" growth rate, which is the growth occurring in the immobilised larvae. Average stress as the mechanoregulatory component of growth did not lead to an accurate simulation of mechanoregulated jaw joint morphogenesis. In contrast, using the differences in the stresses experienced at one side of the joint compared to the opposite side (going from compression to tension) as a stimulus for mechanoregulated growth enabled almost physiological jaw joint morphogenesis to be simulated. The final simulation type, in which only the difference in compressive stresses were used as the mechanobiological stimulus, led to the most physiological growth patterns, suggesting that the local application of compressive stresses are likely to be the main contributors to mechanoregulated growth in the zebrafish jaw joint."

METHODS

Line 375: Anesthetized (immobilized larvae) are presumably not eating. Could lack of nutrients explain lack of growth?

At the stages examined, the larvae are feeding from their maternally deposited yolk sac; therefore there should be no difference in nutritional status between free moving and immobilized larvae. This has been clarified as below.

Page 22, lines 438–440
At the ages studied, larval zebrafish are still dependent on the maternally deposited yolk sack for nutrients so immobilisation would not affect their ability to access nutrients.

Line 380: The method for determining cell growth is not clear without reading the authors' previous J Anatomy paper.

We have expanded our description of the Methods as described below.

Line 388: about how many cells are being analyzed at this timepoint? From Figure1 in the J Anatomy paper, it seems like 15-20 cells?

About 30 cells in 3D, now clarified in the text.

Page 23, lines 458 - 459 :
"Approximately 30 cells constitute the portion of the rudiment modelled, with minimal proliferation over the 24 hour period studied [47]."

Line 391: "The position of cell centroids with respect to each other over time was used to calculated the local rate of deformation." Is this a good measure of growth? What if cells proliferate, migrate without growing, grow in size but centroids stay in the same place, or intercalate? At the time point of interest, 4-5 days, is there any cell proliferation? Are you assuming all growth is due to growth in the size of the cells. Even though this is from the previous paper, it is not well justified in the previous paper either. Provide better assurance that the "statistical velocity gradient" is a good measure of local deformation at this time point.

It is correct that we assume that most growth is from cell expansion, which is justified given that our analyses have shown that proliferation does not play an important role in the day of growth modelled, based on the data from our cell tracking results, and based on the fact that our baseline simulations can predict the real shapes well despite no effects of proliferation being included. The effects of cell intercalation and migration of cells are handled by the code and incorporated into the growth algorithm. We have provided more details on the Methods to address these, and other questions raised in the main text and in the Supplementary data, including equations and graphical explanations.

Figure added (Fig 5)
Supplementary data file D1 added

Changes to Methods :
Page 25, lines 477–492:
"*The position of cell centroids with respect to each other over time was used to calculate the local rate of deformation in cubic regions of interests (ROIs) shown in Fig 5C using the "statistical symmetrised velocity gradient" matrix developed by Graner et al. [63]. Graner et al. [63] defined the statistical symmetrised velocity gradient as a tool to describe at which rate and in which direction the pattern of the tissue deforms between consecutive timepoints t and t+Δt. The gradient quantifies local tissue distortions, such that if neighbouring cells grow or intercalate, or if extracellular matrix is built, the distance between cell centroids, and therefore the geometry of the tissue, change. The statistical symmetrised velocity gradient has units of s^{-1} and is a statistical measurement of tissue changes equivalent to strain rate [63] as described in detail in Supplementary data file (D1). Briefly,*

the relative position of cell centroids at a timepoint t forms a pattern which can be described from the link vectors l *connecting neighbouring cell centroids (Fig 5D). Over time, the link vectors* l *may change in length and direction, changing the pattern's overall geometry (Fig 5D). In our previous paper on free-to-move larvae at the same developmental stage, we showed that growth from 4–5 dpf could be accurately estimated using the velocity gradient methodology [47]. Our prior work also demonstrated that the direct inclusion of cell division was not essential for correct quantification of tissue growth rates and directions, due to the very low rate of proliferation over the time window assessed [47]."*

From the J Anat paper, each cell is about 10 microns and the ROI is 15 microns. How does this work? You are tracking growth of individual cells and mapping this to an ROI? How many ROIs do you have in 3D? How many cells? In this paper, you established a method to identify cells, identify their centroid at each time point, match cells across timepoints. Multiple cells contribute to a statistical velocity gradient, but the "statistical velocity gradient" is not clearly defined (only referenced to another paper). The process of segmenting cells □ obtaining growth directions for ROI is not clear. You need to include enough detail about the "cell dynamics" that we understand what you are doing without having to read multiple previous papers.

We hope that the additional details and figures provided provide clarity on the methods.
Regarding the ROIs, the calculations used the cells within an ROI but also all their neighbours whether or not these were part of the ROI, and this is why the dimensions of the ROIs are greater than the cell diameter. The point has been clarified in the text as below.

Page 25 lines 493–495
"Calculations of the statistical symmetrised velocity gradient were computed for each ROI using the link vectors l *connecting the cells within the ROI to all their neighbours (whether or not these were part of the ROI)."*

Line 395: It is unclear why the cubic ROIs of line 394 suddenly become ellipsoidal ROIs in line 395. Couldn't you represent this growth as axes on a cube?

Further details have been provided in the text to clarify this point.

Page 25 lines 496–500
"*After calculations, the local growth matrix ("statistical symmetrised velocity gradient) can be represented by an ellipsoid whose axes (eigenvectors) represent the three directions for growth and their radius (eigenvalues) the rate of growth along these directions (Fig 5E). Growth maps displaying the local deformation rates and directions were generated from the local growth ellipsoids for each time window (Fig 5F & G)."*

Figure S3. Why aren't VD growth vectors in the VD direction? In day 4.5-5 they are, but in day 4-4.5 some of them seem more AP. If an angle is > 45 wouldn't that indicate that it was being assigned to the wrong axis? It seems like you assigned the directions based on which anatomical axis it was closest to.

The principal growth directions turned out to be more or less aligned with the anatomical directions, but for simplicity in the main text and to not lose the reader we choose a simplified way of presenting the data. We chose to keep the actual growth orientations given by the growth ellipsoids for consistency with the FE models and to ensure that there was no loss of accuracy. Effectively the growth direction are an approximation mainly for reading purposes; clarified in the methods as described below.

Page 26, lines 502–508
For simplicity, ventrodorsal (VD)/anteroposterior (AP)/mediolateral (ML) growth is defined as the growth rates of the growth ellipsoid axis whose angle from the anatomical VD/AP/ML axis is the smallest amongst the three ellipsoid axes. For example, the main direction of deformation (major

axis of the ellipsoid) tended to align with the VD axis, growth rates along main direction of deformation were therefore called VD growth rates. All growth maps were analysed following this terminology for simplicity.

How can you show ML growth in this plane? Why not show it in a plane that shows ML? One would expect all the points to be dots in this view (as seen in the 4.5-5 pdf), which isn't a helpful vector.

Figures have been updated accordingly.

The scale bar in this picture is confusing. It seems like the entire region is about 60 microns long, which from Figure 1 in the J Anat paper is probably about 6 cells. There are way more than 6 cells represented, which makes it seem this is a projection through the entire stack, yet the caption says "results are displayed in one section in the mid-lateral plane".

Each vector is a representation of tissue growth direction, not related to a specific cell. We did try visualising with one vector per ROI but the images were not comprehensible which is why we present the current figure versions.

Line 411: How do you ensure alignment of the images? Cross sectional shape depends on the orientation of the object in the image. Line 417: image stacks were imported, but how were they aligned to be in the same direction?

Images were manually aligned in 3D, based on the characteristic shape of the retroarticular process. Image alignment and orientation is clarified in the text as described below.

Page 23, lines 454–458
"Prior to the cell tracking, all rudiments were aligned in 3-matic (Materialise NV, Leuven, Belgium) at joint line and retroarticular process (shape feature at the ventral side of the MC [62]) and transformation matrices describing this alignment were exported. Transformation matrices which were exported from 3-matic were used to accordingly align the cell centroids of each dataset in MATLAB."

Page 27, lines 524–526
"*The segmented half-jaws were aligned in 3-matic (Materialise NV, Leuven, Belgium) at joint line and retroarticular process (shape feature at the ventral side of the MC [62]). In MATLAB, segmented and aligned half-jaws were […]*"

It seems a shame to represent your 3D images with 2D outlines. There are methods to register 3D shapes and measure differences. Justify the use of 2D outlines.

We did try part comparison in 3D which was more difficult to visualise alignment and was visually unappealing compared to 2D outlines. In 3D, images were also harder to crop, and to visualise the superposition. Therefore, in this case, 2D analyses were superior.

Line 424: This entire section of "measuring modulus" seems fairly irrelevant when you just use a linear elastic model. An 8 micron radius indentor is the same size as the cells. With only 1 micron depth, the contact area is much smaller than the cell. This implies you are just measuring stiffness of a single cell for each of the larvae? Is this really helpful when you end up using a linear elastic modulus? I'd suggest you move this to Supplementary Information, if you want to keep it at all. (I'm not sure measuring stiffness of a single cell is really that relevant to what you are doing.)

We moved this section to supplementary as suggested (supp data file D2)

Line 439: What does "all sections containing relevant cartilage regions" mean? What is a section? What is a relevant cartilage region? It seems like you pressed on one cell in each larva.

This sentence was rephrased for clarity.

"Nano-indentation was performed across all sections in which the cartilaginous regions were observed,"

Line 458: In this section you put growth data from your experiments in your model and it gives back growth that you tell it. This seems like a check to make sure your growth calculations were correct. There are no results from this. You need this section for 2 reasons: 1) use this section to explain clearly how you calculate Gimm and what it means. 2) how you calculate Gfree and that you will use it as a comparison for the simulations (i.e. to "check" your answer).

These sections have been largely rewritten (including moving some text from Supplementary data to the main paper) to address the lack of clarity in the original submission, as detailed below. Details on how growth maps are obtained were added as discussed in a previous comment.

Page 12, lines 212–214
"A computational simulation of MC growth was implemented using finite element (FE) methods. To validate the simulation, we first verified that simulations using the tracked cell activities for either free-to-move or immobilised larvae as the inputs for growth would predict the observed shapes correctly."

Line 482: The loading conditions on the finite element model are not clear. Figure 6 does not indicate the loading conditions applied. Were forces or displacements applied? What was the direction of force? Where was it fixed? How fast was the load applied? Was it applied as a series of quasi static loads or as a dynamic load? Instead of Figure 6, it would be helpful to have the finite element model with the boundary conditions and loading conditions indicated.

More details were provided on the FE models especially on loading and boundary conditions. Fig 6 was updated, with addition to Fig 6B in which the FE model is shown, with boundary conditions and loading conditions.

Page 30, lines 593–596
"Muscle contractions engaged during opening/closure shown in Fig 6 were applied to the models as point loads in fixed directions under quasi-static conditions following a previously published model [64] (Fig 6B). Muscle attachment points and directions were estimated from confocal scans of double transgenic Tg(col2a1aBAC:mCherry;smyhc1:EGFP) larvae (Fig 6Ai)."

Page 28, lines 561–566
"Physiological boundary conditions were applied as previously published [47] and as shown in Fig 6B. The anterior end of the ceratohyal was fixed in all directions (Fig. 6A) and translations in the lateromedial direction of the anterior ends of the Meckel's cartilage were prevented to maintain the symmetry with the missing half-jaw (Fig. 6B). Only anteroposterior translations of the posterior end of the palatoquadrate, where the lower jaw connects to the rest of the craniofacial skeleton, were allowed (Fig. 6B)."

Table 3: How was ratio of muscle forces determined?

Page 30, lines 598–600
"*The ratio of muscle forces was obtained from [19] based on the number of muscles fibres of each muscle engaged during jaw motion measured from confocal scans of double transgenic Tg(col2a1aBAC:mCherry;smyhc1:EGFP) larvae.*"

Line 489: Why do you need stress and strain fields? It is a linear elastic model with homogenous material properties. They'll tell you the same thing. (as you report in line 506). You don't need S6. Just use hydrostatic stress forget the strain.

Appendix S6 was removed as suggested and the main text was updated accordingly.

Line 500: What are "loading fields"?

This terminology was changed to "hydrostatic stress fields"

Line 508: Because the "loading history" is not clear (see above comment), it is not clear how the mechanobiological growth is calculated. How many step increments (N) were applied? This seems to indicate jaw opening and closing is applied as quasi-static. More information on the loading conditions are required. Because you know that bending occurs, it seems rather silly to look at an average hydrostatic stress. The tension and compression experienced throughout opening and closing will cancel each other out. Average does not represent anything (as you found out). If you are only concerned about compression, why would you average it and then only look at compression. It is suggested that solid rationale for this choice of stimulus be made or remove it.

As discussed in a previous comment, more details were provided on the loading and boundary conditions with the addition of Fig 5B in which the FE model is shown.

Page 30, lines 600–602
"Jaw closure and opening were simulated in subsequent steps with each step decomposed into five increments"

Line 516: a is a constant that balances out the influence of mechanics relative to biological growth. Is that correct? You chose it such that the amount of growth matched your experiment?

This is correct. We hope the details below afford more clarity on this variable.

Page 33, lines 632–633
*"a is a mechanoregulatory growth modulating variable which influences the impact of the average hydrostatic stress field **S** on the mechanobiological growth map"*

Page 33, lines 635–640
"A sensitivity analysis was performed to determine the most appropriate value for a. Using the 4–4.5 dpf simulation, a was incrementally increased from 1e9 $m^2N^{-1}s^{-1}$to 4e9 $m^2N^{-1}s^{-1}$. At the lowest value, there was minimal mechanobiological growth, while the maximum value led to obvious joint overgrowth, as shown in Supplementary Figure S5. The modulating variable value which best predicted physiological MC depth growth was chosen (a = 3e9 $m^2N^{-1}s^{-1}$)."

Figure 7 does not clarify anything. It is suggested that you have one figure with the loads/boundary conditions clearly indicated on the FE model. You delete Method 1. You clarify what a hydrostatic stress gradient is. D) "time evolution of hydrostatic stress gradient" is unclear E) This should be one figure showing the hydrostatic stress stimulus in the various planes, clearly labeled. F) not clear what this is showing.

Figure 7 was modified and simplified, providing a clearer picture of was Gimm and Gfree are and on the hydrostatic stress gradients.

Line 545: It is unclear how a stress gradient captures the "dynamic changes in hydrostatic stress". Provide more explicit details on how the gradient was calculated. Are the results different if you just evaluate the maximum hydrostatic compressive stress during a complete

load cycle? Other work has used max hydrostatic compressive stress as a mechanobiological stimulus for decades. It is not clear why this is called "dynamic". It is just at the peaks so just picking up when the gradient is largest. You chose the gradient because you needed a stimulus that varied in the D/V direction. What does hydrostatic stress gradient mean physiologically? What would this translate to at the cell

We provided more details on how the gradients and the mechanobiological maps were calculated as described below.

Page 34, lines 647–667
"Next, we tested the hypothesis that the difference between stress levels at each side of the rudiment compared to its opposite one coupled with the change in this difference over the opening and closing cycle of the joint promotes jaw joint anisotropy. The rational for this hypothesis was that along the ventrodorsal axis, where growth of the joint shape was the most pronounced in normally moving fish, a gradient in stress from tension to compression was observed during jaw opening which was reversed during jaw closure. For each node of the mesh model, the hydrostatic stress gradients $\boldsymbol{\nabla\sigma_h}$ *along each anatomical axes* ***x, y*** *and* ***z*** *were calculated at peak closure and peak opening (the most extreme senarii) in MATLAB [67] (Fig 7C method 2). Here, these hydrostatic stress gradients were expressed in the form of diagonal matrices:*

$$\boldsymbol{\nabla\sigma_h} = \begin{matrix} \frac{\partial\sigma_h}{\partial \boldsymbol{x}} & 0 & 0 \\ 0 & \frac{\partial\sigma_h}{\partial \boldsymbol{y}} & 0 \\ 0 & 0 & \frac{\partial\sigma_h}{\partial \boldsymbol{z}} \end{matrix}$$

where σ_h *is the hydrostatic stress at the considered node and (x, y, z) are the anatomical axes.*

To account for the temporal changes in stress gradients over jaw motion with the hypothesis that a switch in the direction of the gradients promotes growth, changes to the hydrostatic stress gradients between peak opening to peak closure were calculated: the absolute value of the difference between peak opening and peak closure was taken. A new growth map $\boldsymbol{G_{grad}}$ *was calculated at each node of the mesh model based on the following equation:*

$$\boldsymbol{G_{grad}} = \boldsymbol{G_{imm}} + b. \mid \boldsymbol{\nabla\sigma_{h\ peak\ opening}} - \boldsymbol{\nabla\sigma_{h\ peak\ closure}} \mid$$

where $\boldsymbol{G_{imm}}$ *is the growth tensor obtained from immobilised larvae at the considered node, b is a mechanoregulatory growth modulating variable which influences the impact of the hydrostatic stress gradients on the mechanobiological growth map (units* $[m^3N^{-1}s^{-1}]$*) and* $\boldsymbol{\nabla\sigma_{h\ peak\ opening}}$ *and* $\boldsymbol{\nabla\sigma_{h\ peak\ closure}}$ *are the hydrostatic stress gradients at peak opening and peak closure respectively."*

When visualising the maximum hydrostatic compressive stress field, it looked very similar to the average hydrostatic stress field (except with a change to the order of magnitude) as shown below. Running simulations with this stimulus would likely be very similar to our average simulations so we decided to not include this stimulus to the current work.

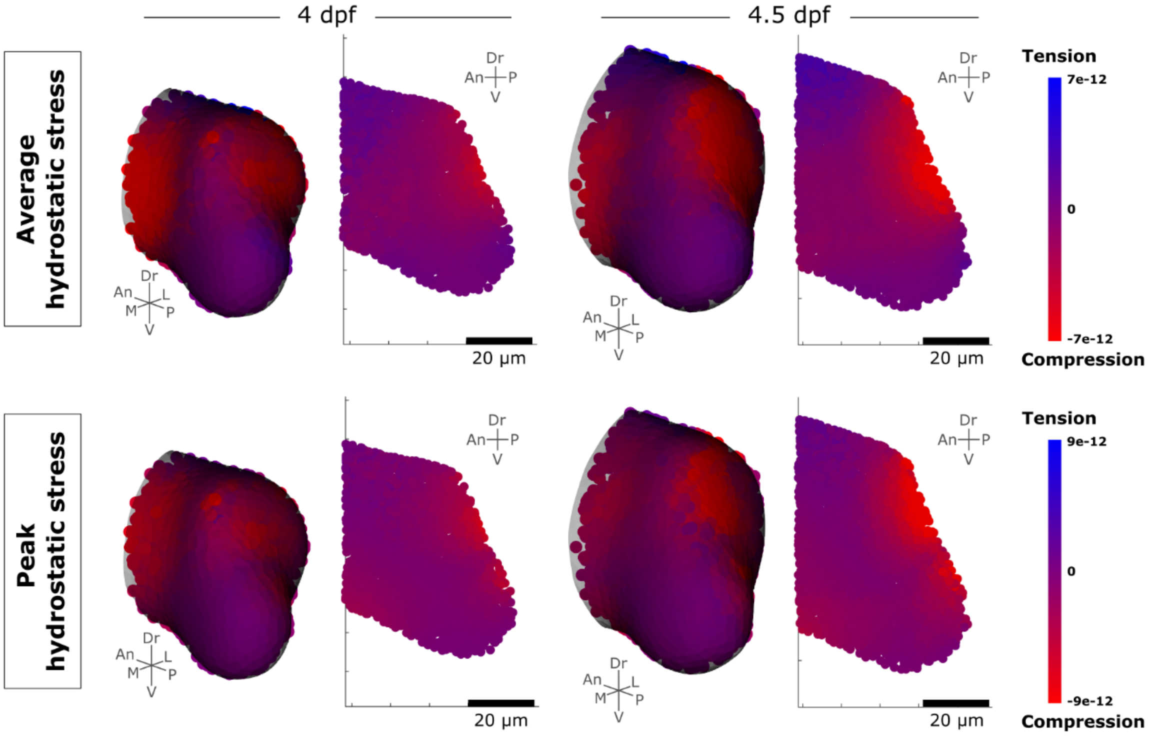

Hydrostatic stress gradient is pressure gradient, cells sense a trigger from only one side (or different triggers from two opposite sides), which may promote directed behaviours of the cells: change of orientation, allometric volume expansion, directed proliferation, etc, which may in turn explain this growth anisotropy (especially ventral to dorsal).

Page 20, lines 382–395
The concept of a difference in biophysical stimuli within different parts of an organ (i.e., a gradient) affecting developmental change has been proposed as influencing epithelial function and pathology [51], while new bone formation in an animal model of increased loading was positively correlated with the spatial distribution of fluid pressure gradients [52]. Our combined morphological, cell-level and mechanobiological simulation analyses reveal that the growth anisotropy in the zebrafish jaw joint can only be promoted by an anisotropic growth stimulus, such as the hydrostatic gradient tested in the present work. We theorise that pronounced changes to and from high compression levels promotes fluid flow and physical deformation of the cells, leading to mechanobiological change. From our simulations of mechanoregulated growth we also conclude that tension is not a large contributor to joint growth anisotropy. The behaviour of the interzone in the un-cavitated joint as defined in our models may not be physiologically representative, and it is possible that a more accurate representation of the interzone properties would alter the distributions of compression and tension. However, our study clearly identifies the importance of anisotropic growth stimuli for anisotropic growth of the zebrafish larval jaw joint.

Line 549: Is this the absolute value of the difference of the gradients? Things are getting confusing. Is it really a time evolution if you just look at peak opening and closing?

Having noticed the inversion between compression and tension in hydrostatic stress field between peak opening and peak closure, we calculated the gradient based on peak opening and peak closure in the mechanobiological growth maps as the most extreme scenarios. We provided more details on how the gradients and the mechanobiological maps were calculated as discussed in the previous comment.

Line 555: Check units of the constant. Do you need sec in there?

Mistakes were made in units and in coherence of equations, now fixed.

Line 565: This is unclear. Did you calculate the absolute value of the gradients of only the compressive stresses? Is Figure 7, Method 2 showing the stress gradients in the x,y directions? It would help if you provide a figure (can be in Supplemental) where you show the hydrostatic stress in the two planes of interest (like Figure 4B, but showing the M/L plane as well), then the hydrostatic stress gradients in the 3 directions (2 can be shown in one plane and M/L in another plane), then the hydrostatic compressive stress gradient in the 3 directions. This would really help show what is happening with the manipulations. This likely means adding to figure 4. 4A can be discarded unless you have a good rationale for trying this.4B is likely max tension/compressive stress. Add to this the gradients in the different planes so we can see the stimulus as contour plots.

Additions to the Introduction, Results and Methods as discussed in previous comments should afford more clarity on the processes used in this research. For further clarity, Figure 7 was rearranged and honed to help the reader. We hope that the extensive revisions to the text and figures as detailed above have made the concept of the gradients of stresses more clear.

Table 4: Remove “time evolution”. This is not an accurate descriptor.

This terminology was removed.

__**Reviewer #2:**__

This work presents an interesting multidisciplinary work, where experiments and computational simulations are combined in order to identify links between the mechanical stimuli arising from movement and patterns of growth.

Although the work is really very well presented and very interesting, there are some relevant aspects that require further explanation or justification:

1) Authors indicate in the paper that they use an integrated cell-level data into a novel mechanobiological model. It is not clear for me what they mean "an integrated cell-level data".

We rewrote the end of the Introduction to afford more clarity on our methods and throughout the main text we removed the terminology of “integrated cell-level data” to instead describe our models with more details as described below.

Page 7, lines 132–143
“We then quantified the dynamics of growth underlying normal or immobilised growth by tracking individual cells over the developmental period studied, and found that growth rates were most diminished by larval immobility along the ventrodorsal axis. Next, we implemented a mechanobiological simulation of jaw joint morphogenesis and validated that simulations using the tracked cell activities for free to move and immobilised larvae as the inputs for growth predicted the observed shapes correctly. Finally, we implemented simulations in which immobilised growth rates were applied to control joint shapes (serving as the baseline biological contribution to growth), supplemented by a mechanobiological component of growth in order to test hypotheses relating different types of mechanical stimuli to joint morphogenesis. Using this methodological approach, we tested which mechanical stimuli arising from jaw movements are most likely to direct mechanoregulated growth anisotropy in the zebrafish jaw joint.”

2) Authors conclude that the application of cyclic compression, rather than the cyclical switch from compression to tension, is likely to be the key contributor to jaw joint morphogenesis. Why do you think it is happening? Could it be due to the fluid flow associated to the compression loads? I think authors should discuss further why they think which is the local mechanobiological effect that could be more relevant for joint morphogenesis.

Thank you for this excellent suggestion, which we have incorporated into the Discussion as detailed below. We have also clarified our terminology throughout as tracked and also as described in the response to Reviewer 1.

Page 20, lines 382–395
The concept of a difference in biophysical stimuli within different parts of an organ (i.e., a gradient) affecting developmental change has been proposed as influencing epithelial function and pathology [51], while new bone formation in an animal model of increased loading was positively correlated with the spatial distribution of fluid pressure gradients [52]. Our combined morphological, cell-level and mechanobiological simulation analyses reveal that the growth anisotropy in the zebrafish jaw joint can only be promoted by an anisotropic growth stimulus, such as the hydrostatic gradient tested in the present work. We theorise that pronounced changes to and from high compression levels promotes fluid flow and physical deformation of the cells, leading to mechanobiological change. From our simulations of mechanoregulated growth we also conclude that tension is not a large contributor to joint growth anisotropy. The behaviour of the interzone in the un-cavitated joint as defined in our models may not be physiologically representative, and it is possible that a more accurate representation of the interzone properties would alter the distributions of compression and tension. However, our study clearly identifies the importance of anisotropic growth stimuli for anisotropic growth of the zebrafish larval jaw joint.

3) From the FE-based simulations, it is not clear for me which kind of elements and materials have been used for the simulations. In my opinion, the FE model should be described providing much more data, such as, the type of element used, the constitutive law used, boundary conditions and interaction constraints.

We have provided more details on the FE models especially on loading and boundary conditions on the Methods (section "Simulating zebrafish jaw joint morphogenesis") in the main text and with graphical explanations with the addition of Fig 6B in which the FE model is shown, with boundary and loading conditions.

Page 27, lines 543–545
"Morphologies were meshed in 3-matic with ten node tetrahedral elements of approximate size 2 µm In Abaqus CAE, a finite element (FE) model for the starting point of each twelve-hour time window was created from the meshed assemblies."

Page 28, lines 561–566
"Physiological boundary conditions were applied as previously published [47] and as shown in Fig 6B. The anterior end of the ceratohyal was fixed in all directions (Fig. 6A) and translations in the lateromedial direction of the anterior ends of the Meckel's cartilage were prevented to maintain the symmetry with the missing half-jaw (Fig. 6B). Only anteroposterior translations of the posterior end of the palatoquadrate, where the lower jaw connects to the rest of the craniofacial skeleton, were allowed (Fig. 6B)."

Page 30, lines 593–596
"Muscle contractions engaged during opening/closure shown in Fig 6 were applied to the models as point loads in fixed directions under quasi-static conditions following a previously published model [63] (Fig 6B). Muscle attachment points and directions were estimated from confocal scans of double transgenic Tg(col2a1aBAC:mCherry;smyhc1:EGFP) larvae (Fig 6Ai)."

Page 30, lines 600–601
"Jaw closure and opening were simulated in subsequent steps with each step decomposed into five increments"

<u>Reviewer #3:</u>

The goal of this work was to directly identify the relationship between biomechanical stimuli due to embryonic movement and joint morphogenesis using zebrafish jaw as a model system to directly measure cell activity and mechanical environment, and implemented these measures in a mechanobiological model of jaw morphogenesis. This paper offers a very strong background and motivation for quantification of biological stimuli and spatial analyses to augment understanding morphogenesis of joints and biomechanical predictive models of this process. The methods are appropriate and overall this is a very strong contribution to the literature.

We thank the reviewer for their positive feedback.

The comparison between the dynamic simulations with and without inclusion of the tensile contribution is interesting. Because the simulations that do not consider tension capture the morphological development better, the authors conclude that the tension is not a large contributor. However, the tension appears to be a real condition experienced by the developing jaw, even though including it in the model leads to worse predictions – this could equally be a result of some error in the model itself in terms of how tensile stimuli are represented. Could the authors expand on this point in the discussion?

It is indeed possible that the tensile stresses predicted in our FE models may not be accurately represented, due to some uncertainty in the properties of the interzone. Our models of the joint cover a period where the joint is not yet cavitated but it is possible that the interzone allows more flexion or movement than we have allowed in the simulation, which would reduce the element of bending conditions. We have included a discussion of this point as detailed below.

Page 20, lines 391–395
The behaviour of the interzone in the un-cavitated joint as defined in our models may not be physiologically representative, and it is possible that a more accurate representation of the interzone properties would alter the distributions of compression and tension. However, our study clearly identifies the importance of anisotropic growth stimuli for anisotropic growth of the zebrafish larval jaw joint.
